# The effects of platelet rich plasma and zinc oxide nanoparticle on skin wound healing in dogs

**DOI:** 10.1038/s41598-026-54633-7

**Published:** 2026-06-02

**Authors:** Mona N. Wafy, Elham A. Hassan, Samar Saeed, Marwa S. Khattab, Huda O. AbuBakr, Ashraf M. Abu-Seida

**Affiliations:** 1 Department of Surgery, Anesthesiology and Radiology, Faculty of Veterinary Medicine, Cairo University, Giza, 12211 Egypt; 2 National Institute of Laser Enhanced Sciences, Cairo University, Giza, 12613 Egypt; 3 Department of Pathology, Faculty of Veterinary Medicine, Cairo University, Giza, 12211 Egypt; 4 Department of Biochemistry and Molecular Biology, Faculty of Veterinary Medicine, Cairo University, Giza, 12211 Egypt; 5 Department of Biochemistry, Faculty of Veterinary Medicine, Egyptian Chinese University, Cairo, Egypt; 6 Animal Research Facility, Galala University, Suez, Egypt

**Keywords:** Dogs, Epithelialization, Matrix extracellular phosphoglycoprotien, Tumor necrosis factor alpha, Transforming growth factor beta, Wound contraction, Biotechnology, Cell biology, Diseases, Medical research

## Abstract

Wound healing is a complicated process, so it’s critical to identify efficient ways to hasten recovery. Platelet-rich plasma (PRP) and zinc oxide nanoparticles (ZnO NPs) have demonstrated potential in improving cutaneous wound healing in a variety of species. But little is known about their combined effects, especially in dogs. Therefore, this study determined how topical infiltration of PRP and ZnO NPs ointment, both separately and in combination, affect the healing of dogs’ cutaneous wounds. Thirty-six full skin wounds were induced in the chest of six adult mongrel dogs. These wounds were randomly divided into six equal groups (6 wounds each) according to treatment protocol: group 1 served as a control and the wounds were dressed daily with normal saline only, group 2: the wounds were dressed daily with lanolin only, group 3: the wounds were infiltrated once with PRP, group 4: the wounds were treated with PRP single infiltration combined with lanolin ointment daily dressing, group 5: the wounds were dressed daily with ZnO NPs ointment, and group 6: the wounds were infiltrated once with PRP and daily dressed with ZnO NPs ointment. Wound healing progress was monitored; epithelialization, wound contraction, and overall healing were assessed. Total antioxidant capacity (TAC), malondialdehyde (MDA) and the concentration of platelets derived growth factor beta (PDGFβ) were measured on wound fluid. Gene expression of matrix extracellular phosphoglycoprotien (MEPE), transforming growth factor beta (TGF-β) and tumor necrosis factor alpha (TNF-α) were also evaluated on skin biopsies at day 0, 5, 10 and 20. Histopathology, immunohistochemistry and staining of collagen bundles were performed on skin biopsies at 5, 10 and 20 days of wound induction. All data were statistically analyzed. There was a significant interaction between the group and time across all parameters (*P* < 0.001). The PRP–ZnO NPs group consistently has a great effect on wound size reduction, contraction, healing, epithelialization, and antioxidant activity, along with higher MEPE and PDGFβ expression and arranged parallel collagen bundles, indicating enhanced regeneration. While PRP alone showed the strongest TGF-β increase and anti-inflammatory effect (lowest TNF-α). PRP–ZnO NPs provided the best overall balance between regeneration and inflammation control. All treatments surpassed the control and lanolin groups, which showed minimal improvement. PRP–Lanolin and ZnO NPs offered moderate benefits but were less effective than PRP–ZnO NPs or PRP. ZnO NPs and PRP work together to improve skin wound healing in dogs; PRP promotes regenerative signaling, while ZnO NPs reduce oxidative stress and microbial load.

## Introduction

The four stages of the complex physiological process that repairs injured tissue and heals wounds are hemostasis, inflammation, proliferation, and remodeling. It involves a number of biological processes, including as blood clotting, fibroblast collagen deposition, tissue rearrangement, and immune cell infiltration to prevent infection. Appropriate wound care, including cleaning and dressing, is necessary to promote healing^1^.

Dogs frequently have cutaneous wounds, which can result from trauma, surgery, or medical disorders^2^. Hemostasis, inflammation, proliferation, and remodeling are all steps in the intricate, multi-stage biological process that heals these wounds. To completely restore the integrity of the skin, each phase necessitates a coordinated response including different cell types, cytokines, and growth factors^3^. However, a number of obstacles, including inflammation, bacterial infection, and poor re-epithelialization, can hinder this healing process, resulting in chronic wounds or delayed wound closure^4,5^.

Effective antibacterial activity in blood platelets promotes granulation tissue development, re-epithelialization, and wound healing in infected wounds^6^. Platelet-derived growth factor (PDGFβ), transforming growth factor-β (TGF-β), vascular endothelial growth factor (VEGF), and platelet-derived angiogenesis factor (PDAF) are among the many wound healing agents that the blood platelets are a wonderful source of^7^. The healing process is enhanced, reinforced, and accelerated when the concentration of platelets in the wounded area is increased^8^. Accordingly, to promote tissue regeneration and enhance the healing of complex wounds, PRP serves as a scaffold and source of growth factors^9,10^.

A promising alternative for quicker wound healing is nanomaterials^11^. Through its antibacterial, anti-inflammatory, and angiogenic properties, it can activate a variety of cellular and molecular mechanisms that regulate the wound microenvironment^12^. Because of their high surface area to volume ratio and nanoscale size, nanomaterials have special properties. They can also undergo nanosizing in conjunction with changes in their physical and chemical properties^13–15^. New avenues for enhancing wound healing have been made possible by the use of nanotechnology.

The special qualities of ZnO NPs, such as their antibacterial, antioxidant, and anti-inflammatory qualities, have drawn a lot of interest. Because of these qualities, they are useful for accelerating wound healing while also boosting tissue regeneration and avoiding infection^16,17^.

According to previous studies, ZnO NPs can promote angiogenesis, collagen synthesis, and cell proliferation, all of which are essential for successful wound healing^18^. Notwithstanding these encouraging results, there are still issues with optimizing nanoparticle formulations to reduce toxicity and increase efficacy. Studies have indicated that the incorporation of ZnO NPs into natural matrices or biocompatible polymers can improve their therapeutic benefits and safety. For instance, it has been discovered that adding ZnO NPs to hydrogels or composite materials improves antibacterial activity and biocompatibility while lowering possible cytotoxicity^19,20^. Furthermore, recent research has demonstrated that ZnO NPs, when applied topically, promote re-epithelialization and speed wound contraction without causing serious adverse effects^21^.

Given the growing concern over antibiotic resistance in veterinary medicine, ZnO NPs’ potential to reduce infection and inflammation while accelerating wound closure is especially pertinent. ZnO NPs may be a good substitute or supplement to traditional therapies, helping animals’ cutaneous wounds heal more quickly and effectively^22–24^.

In order to assess wound healing treatments in a physiologically integrated setting that cannot be duplicated in vitro, animal models are still crucial. Compared to laboratory animal models such as rats and mice, which mostly heal via contraction due to the panniculus carnosus, larger animal models provide more clinically relevant healing patterns. Dogs are used as an experimental model because of their physiological similarities to human skin regeneration systems and their established significance in wound healing research. Canine wounds exhibit similar healing processes, such as granulation tissue formation and epithelialization, which are essential for translational research^25^. Our study’s objective was to assess how ZnO NPs ointment applied daily and PRP single local infiltration affect the healing of dog’s cutaneous wounds.

## Materials and methods

### Preparation of ZnO NPs

After adding 1.5 g of zinc acetate to 0.9 g of sodium hydroxide and continuing the mechanical grinding process, 0.9 g of sodium alginate was added, mechanically blended, and ground for 5 min at room temperature. To obtain pure powder of ZnO NPs stabilized or coated with sodium alginate, the powder was washed with distilled water and then put through a centrifugation step. Lastly, a freeze-drying device was used to dry the wet, fine ZnO NPs powder^26^.

### Characterization of ZnO NPs

#### Scanning electron microscopy (SEM)

The SEM images were obtained using ZEISS FE-SEM ULTRA Plus (equipped with an EDX analyzer) microscope with Philips CM20 microscope, operating at an accelerating voltage of 200 kV.

#### Fourier transform infrared spectrophotometer (FTIR)

A FTIR spectrophotometer (Jasco FT/IR 460 plus spectrometer, Tokyo, Japan) fitted with an attenuated total reflection (ATR) cell was used to obtain the FTIR of the ZnO NPs. After being compressed into pellets using KBr, the samples were added to the ATR crystal. The FTIR spectra were then scanned at room temperature with a resolution of 4 cm^− 1^ and a speed of 2 mm^− 1^ in the wavelength range of 500–4000 cm^− 1^.

#### X-Ray Diffraction (XRD)

Cu Ka radiation (k = 1.54186 A°) was used in XRD measurements using a Philips PW1710 X-ray diffractometer. With a step size of 0.020° 2 H, the XRD patterns were recorded from 5° to 80° 2 H, collecting 10 s per step.

### Preparation of ZnO NPs ointment

Two grams of ZnO NPs were first dissolved in 8 mL of N-Methyl-2-pyrrolidone (NMP) solvent in order to create the ZnO NPs ointment. To guarantee total dissolution, the ZnO NPs were moved to an appropriate container, the NMP solvent was added, and the mixture was sonicated. Separately, 90 g of lanolin were moved to a glass container, and melted into a uniform liquid in a sonicator or water bath. When the melted lanolin and ZnO solution were ready, the lanolin was carefully added to the ZnO mixture, which was then vigorously stirred to guarantee that the nanomaterial was distributed evenly throughout the lanolin base. The final mixture turned into an ointment, which was subsequently put into storage containers. In order to improve texture, the containers were frozen for about five minutes to help them solidify. To preserve stability and effectiveness, the finished product was kept out of direct sunlight in a cool, dry location.

### Preparation of autologous PRP

A 10 mL whole blood sample was taken from the cephalic vein of each dog in a citrate-phosphate dextrose solution and centrifuged at 1500 rpm for 10 min. There were three layers after centrifugation. The plasma layer at the top was transferred to another centrifuge tube and centrifuged again at 3000 rpm for 20 min, yielding two layers: platelet-poor plasma (PPP) at the top and PRP below. After that, calcium chloride was added to the PRP in a 1:10 ratio (0.1 ml CaCl_2_ for every 1 mL PRP) for activation^9^. One mL of activated PRP was immediately injected subcutaneously into multiple wound sites to avoid plasma jellification^27^.

### Evaluation of PRP

Each dog’s total blood count was measured. After PRP was generated, the platelet count was calculated from the PRP sample using Mindary Vet BC 5000 (Mindray Animal Medical Technology Co., LTD Country of Origin: China). Also the samples were evaluated for the presence of WBCs and RBCs.

### Experimental animals

The current study was approved by the Institutional Animal Use and Care Committee at Faculty of Veterinary Medicine, Cairo University, Egypt. Additionally, the Animal Research: Reporting in Vivo Experiments (ARRIVE) guidelines were adhered to. All methods were carried out in accordance with the relevant guidelines and regulations.

This study used six adult healthy mongrel dogs that were purchased commercially from Al-Fahad Trading Company for Animals (Abu-Rawash, Giza, Egypt). The weight and age of these dogs ranged between 16 and 18 kg and one and two years, respectively. Every dog received a comprehensive medical examination, a full blood count, and a serum biochemistry analysis before the trial began. Prior to the experiment, the dogs were housed in different kennels measuring 1.5 m X 2.5 m X 3 m, and they were given two weeks to get used to their new surroundings and diet. They were kept in standard conditions with a 12-hour light/12-hour dark cycle, 25 ± 2 °C temperatures, 60 ± 5% humidity, and proper ventilation. They were provided free access to water and fed commercial maintenance dry food twice a day. The period of the in-vivo experiment was three weeks (May 2024).

### Study design

The six dogs randomly underwent three bilateral full thickness skin wounds on their chests, for a total of 36 wounds. In accordance with the treatment protocol, these wounds were divided randomly into six groups (6 wounds each) as follows:

Group 1 (Control group): the wounds were daily dressed with normal saline.

Group 2 (Lanolin group): the wounds were daily dressed with lanolin only.

Group 3 (PRP group): the wounds were locally infiltrated with single autologous PRP injection.

Group 4 (PRP-Lanolin group): the wounds were locally infiltrated with single PRP injection and dressed daily with lanolin.

Group 5 (ZnO NPs group): the wounds were dressed daily with ZnO NPs carried on lanolin in the form of ointment.

Group 6 (PRP-ZnO NPs): the wounds were locally infiltrated with single PRP injection and dressed daily with ZnO NPs ointment.

After the experimental period, all dogs were treated and released.

### Surgical procedures

Dogs were premedicated with 0.05 mg/kg atropine sulphate (Atropine^®^, Sunways Pvt. Ltd., Mumbai, India) subcutaneously and 1 mg/kg xylazine HCl 2% (Xylamed^®^, Bimeda Animal Health, Dublin, Ireland) intramuscularly. Anesthesia was induced using 10 mg/kg ketamine HCl 5% (Ketalar^®^, JHP pharmaceuticals, Michigan, USA) intramuscularly and maintained with 25 mg/kg thiopental sodium 2.5% solution (Thiopental sodium^®^, Livealth Biopharma Pvt., Ltd., Mumbai, India) given intravenously.

The dogs were positioned on the sternum, and bilateral areas of the dorsal midline were prepared for aseptic surgery by clipping, shaving, and disinfecting with povidone iodine solution. Three circular full-thickness skin wounds, each with a 3 cm diameter, were induced bilaterally, 5 cm from the dorsal midline in the thorax region^28^. Hemostasis was achieved with sterile gauze tampons. All wounds were covered with sterile dressing, gauze, and elastic bandage. Elizabeth collars were used to prevent interference with the wounds. Dogs were given carprofen (4 mg/kg) orally once daily for up to three days post-surgery to manage pain. The induced wounds were then dressed daily according to the group.

### Objective evaluation of wound healing

The diameter of each wound was measured with a digital caliper on day zero. Measurements were repeated on days 7, 14, and 21. Macroscopic images of the wounds were captured using a digital camera at the same intervals. A fixed camera system, regulated illumination, and a constant distance of 20 cm were used to take standardized wound photos. Every image had a metric scale for calibration. Prior to the administration of therapy, the animals were positioned consistently, and pictures were taken at predetermined intervals. Wound size was estimated using standardized techniques by taking photos and measuring the wound using the software digimizer^29^.

The wound healing process was tracked on a daily basis, with documentation of any changes in wound appearance, such as inflammation, granulation tissue production, and epithelialization. Observations regarding pain, discomfort, or signs of infection in the dogs were also recorded.

The percent of wound healing in all groups and the percent of epithelization were calculated according to the following equations:

Percent of wound size (%WS) at day (n) = TWA (n)/ TWA (zero) * 100.

Percent of wound contraction (% WC) at day (n) = 100 - % of wound size (n).

Epithelization percent (%E) at day (n) = Epithelization area (n)/ TWA (n) *100.

Percent of non-healing at day (n) = granulation area (n)/ TWA (zero) * 100.

Percent of healing at day (n) = 100 - % of non-healing (n) according to (Soares et al.^30^. Where: (n) = specific day and (zero) = day 0.

All variables were measured four times, at day 0, 7, 14, and 21 of wounds induction.

### Preparation of wound fluid

A standardized technique for collecting wound fluid was conducted at the clinical site following a previously outlined protocol at day 0, 5, 10 and 20^9^. In summary, the skin wounds were cleansed with sterile water before applying an occlusive dressing over the wound^9^. After 10 min, exudates that accumulated under the dressing were collected by washing with 1 ml of saline solution. The collected wound fluid samples were then centrifuged at 14,000 × g for 10 min. The protein content of each sample was determined according to the method described before^30^. Aliquots were prepared from the samples and stored at -80 °C until further analysis. These wound fluid samples were utilized for evaluating the TAC and the lipid peroxidation marker.

### Assessment of oxidative stress biochemical markers in different groups

#### Assessment of total antioxidant capacity (TAC) (mM/L)

Total antioxidant capacity was determined by enzymatic reaction of samples antioxidants capacity against residual H_2_O_2_ that involved the conversion of 3, 5, dichloro − 2– hydroxy benzensulphonate to a colored product. The absorbance of colored product was measured at 505 nm by UNICO-UV-2100 spectrophotometer according to Koracevic et al.^31^.

#### Assessment of malondialdehyde (MDA) concentration (nM/ml)

Malondialdehyde concentration was used as an index of lipid peroxidation as described by Ohkawa et al.^32^. MDA was determined by measuring the thiobarbituric acid reactive species via diagnostic kits (Bio Diagnostic kits, Giza, Egypt). The absorbance of the colored product was measured at 505 nm by UNICO-UV-2100 spectrophotometer^31^.

### Preparation of skin biopsies

Skin biopsies were prepared by rinsing the surface of each wound with normal saline before treatment began and then biopsies were obtained at day 0, 5, 10, and 20 from the wounds in all groups under rigorous aseptic conditions. Samples were obtained using an 8-mm biopsy punch. Samples were taken from the wound margin including the defect as well as 2–3 mm of normal skin with a depth of 4 mm and stored at -80 °C until gene expression analysis.

### Gene expression analysis: Quantitative real-time polymerase chain reaction (qPCR) for matrix extracellular phosphoglycoprotirn (MEPE), transforming growth factor-beta (TGF-β) and tumor necrosis factor-alpha (TNF-α)

Total RNA was extracted from skin biopsies using QIAmp RNA mini kit (QIAGEN, Hilden, Germany) as indicated by the manufacturer. Total RNA purity and concentration were obtained using a nanodrop ND-1000 spectrophotometer. The isolated RNA was used for cDNA synthesis using reverse transcriptase (Fermentas, EU). Real-time PCR (qPCR) was performed in a total volume of 20 µl using a mixture of 1 µl cDNA, 0.5 mM of each primer **(**Table 1**)**, iQ SYBR Green Premix (Bio-Rad 170–880, USA). PCR amplification and analysis were achieved using Bio–Rad iCycler thermal cycler and the MyiQ realtime PCR detection system. Each assay included triplicate samples for each tested cDNAs and no-template negative control; the expression relative to control was calculated using the Eq. 2-ΔΔCT^33^.

**Table 1:** Primer sequences of reference gene glyceraldhyde-3-phosphate dehydrogenase (GAPDH), Transforming growth factor-Beta (TGF-β), Tumor necrosis factor alpha (TNF-α) and matrix extracellular phosphoglycoprotein (MEPE) genes of Canis lupus familiaris.

Target genes	Accession no.	Sequence (5’ to 3’)	Product size
**GAPDH** **(Reference gene)**	XM_038448971.1	**F: 5’- ATGGGCGTGAACCATGAGAA − 3’** **R: 5’ CAGTGGAAGCAGGGATGATGT-3’**	238 bp
**TNF-α**	NM_001003244.4	**F: 5’ - GCCTCTTCTCCTTCCTCCTC − 3’** **R: 5’ - TGTCACTTGGGGTTCGAGAA − 3’**	159 bp
**TGF-β**	XM_038656896.1	**F: 5’- TCAAGAAAAGTCCGCACAGC − 3’** **R: 5’ - GCGCCAGGAATCATTGCTAT-3’**	170 bp
**MEPE**	NM_001313825.1	**F: 5’- TCTTTTCAGCGTGACTTGGGCA − 3’** **R: 5’- AGGTGCTGGCTCTTGATTTCTTCT − 3’**	247 bp

### Measuring the level of canine platelets derived growth factor-bête (cPDGFβ)

Wound fluid PDGF levels were measured using a canine platelet growth factor subunit B, PDGFβ ELISA Kit (Catalog No: BZEK1862, Chongqing Biospes Co., Ltd, China) according to the manufacturer’s instructions. The optical density of the samples was recorded at a wavelength of 450 nm using a microplate reader (ELx800TM Absorbance Readers, BioTek Instruments, Inc., Vermont, USA). The sample concentration was calculated through the straight-line regression equation of the standard curve of the standard concentration and the OD value, with the sample OD value in the equation OD = a* Conc. + b where OD (Optical Density or Absorbance) is the measured absorbance of a sample at a specific wavelength, Conc. (Concentration): The concentration of the substance being measured, a (Slope): The proportionality constant that relates absorbance to concentration (determined from a calibration curve), b (Intercept): The absorbance value when the concentration is zero (background absorbance)^34^.

### Sample preparation for histopathology evaluation of skin wounds

Tissue specimens of the skin wounds at 5-, 10- and 20-days post induction were fixed in 10% neutral buffered formalin and then processed by paraffin embedding technique. 4 μm thick tissue sections were made using rotary microtome and stained by hematoxylin and eosin stain and Masson’s trichrome (MTC). A light microscope installed with digital camera was used for examination. The positive area % of MTC stained collagen was measured using (image-J software Developer: National Institutes of Health (NIH), USA, Website: https://imagej.nih.gov/ij/) in 3 captured images/wound at 100 X magnification power.

### Immunohistochemical analysis

Tumor necrosis factor alpha (TNF-α) was evaluated in wounds at 5-days post induction. Anti-TNF-α (1:100, Santa Cruz, USA) was applied to de-parafinized tissue after antigen retrieval using citrate buffer PH 6. Universal Immuno-Detector DAB HRP Brown Detection System (Bio SB, USA) was then applied on slides according to manufacturer protocol. The positive area % of TNF- α was measured using image-J software in 3 captured images/wound at 200 X magnification power.

### Statistical analysis

The data have been analyzed by the IBM SPSS Statistics 26 (IBM Corp, Armonk, NY, USA) program. The descriptive statistics were displayed in mean, and standard deviation for quantitative variables and percentage for the qualitative variables. Repeated measure ANOVA was used to find the significant difference in the effect of different protocols on the chemical and physical indices of wound healing including: wound size percent, wound contraction percent, wound healing percent and epithelization percent, The chemical indices of wound healing including, TAC, MDA) concentration, Matrix MEPE, TGF-β, TNF-α and PDGFβ.

Furthermore, one-way ANOVA was used to find the significant difference between groups for each dependent variable, and if the results were significant Post Hoc Tukey test was used to find the result for between-group comparisons. All these dependent variables were measured four times respectively. T test was used. The statistical significance was set at *P* < 0.05.

## Results

### Findings of SEM

**Fig. 1:**
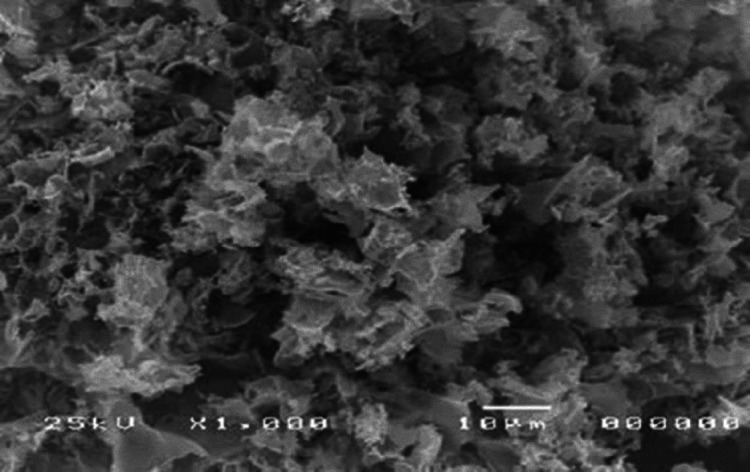
SEM image of the prepared ZnO NPs.

The ZnO NPs dispersed coarsely and interlink with each other forming the ZnO flower structure **(**Fig. 1**).** There was no agglomeration of each constituent, which proved the nano properties of the synthesized materials.

### Findings of FTIR

**Fig. 2:**
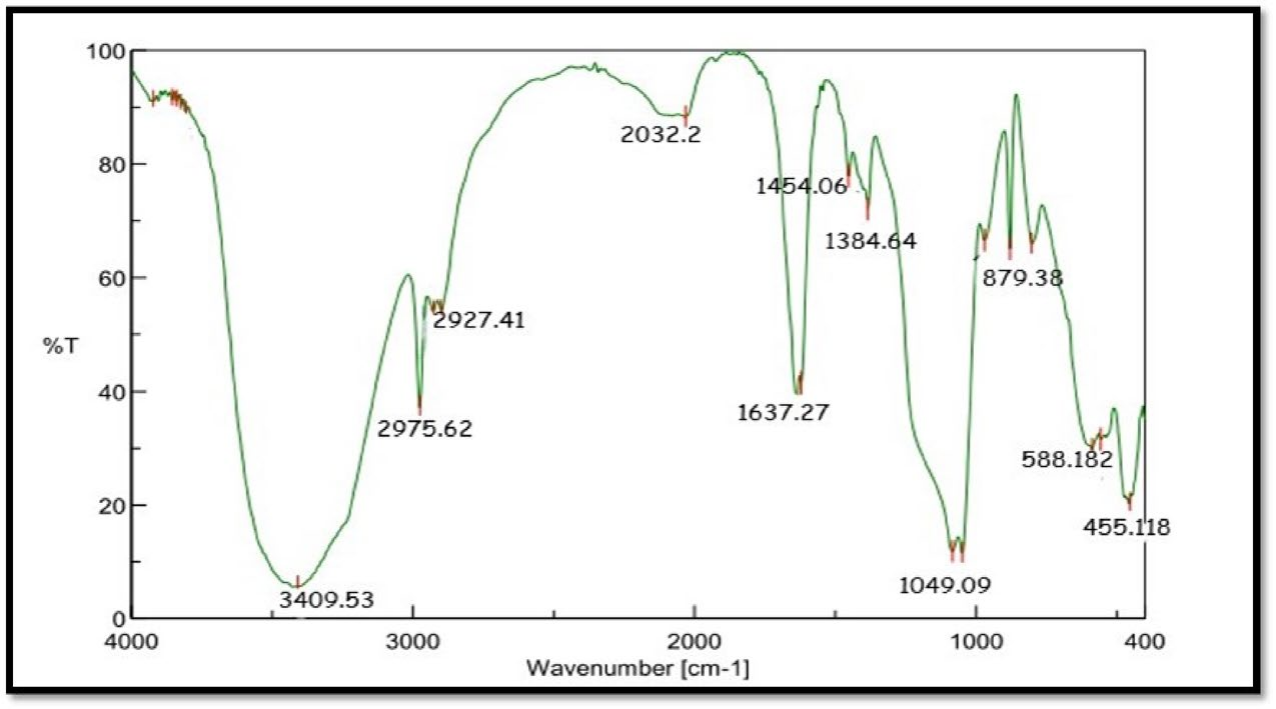
FTIR image of the prepared ZnO NPs.

The functional group of ZnO NPs was indicated using the peaks. The samples had 3409.53, 1637.27, 1384.64, 1049.09, and 879.38 absorption peak. The absorption peak at 3409.91 corresponded to O–H stretch and 1637.27 corresponded to C-O stretch **(**Fig. 2**).**

### Findings of XRD

**Fig. 3:**
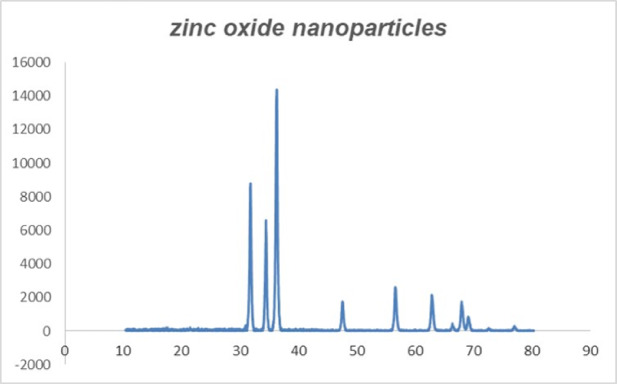
XRD image of the prepared ZnO NPs.

The XRD patterns of the prepared ZnO NPs are shown in Fig. 3. Diffraction peaks corresponding to the impurity were not found in the XRD patterns, confirming the high purity of the synthesized products. The XRD peaks at 31.70°, 34.38°, 36.25°, 47.50°, 56.50°, 60.0°, 66.60°, 69.1°, and 70.48° were identified as (1 0 0), (0 0 2), (1 0 1), (1 0 2), (1 1 0), (1 0 3), (2 0 0), (1 1 2), and (2 0 1) reflections, respectively.

### Results of PRP evaluation

The mean blood platelets count was 277 ± 32 × 10^3^/µl while the mean platelet count in the prepared PRP was 896 ± 45 × 10^3^/µl. The PRP samples showed very little number of WBCs (50 ± 10/µl) and RBCs (100 ± 20/µl).

### Clinical findings

**Fig. 4:**
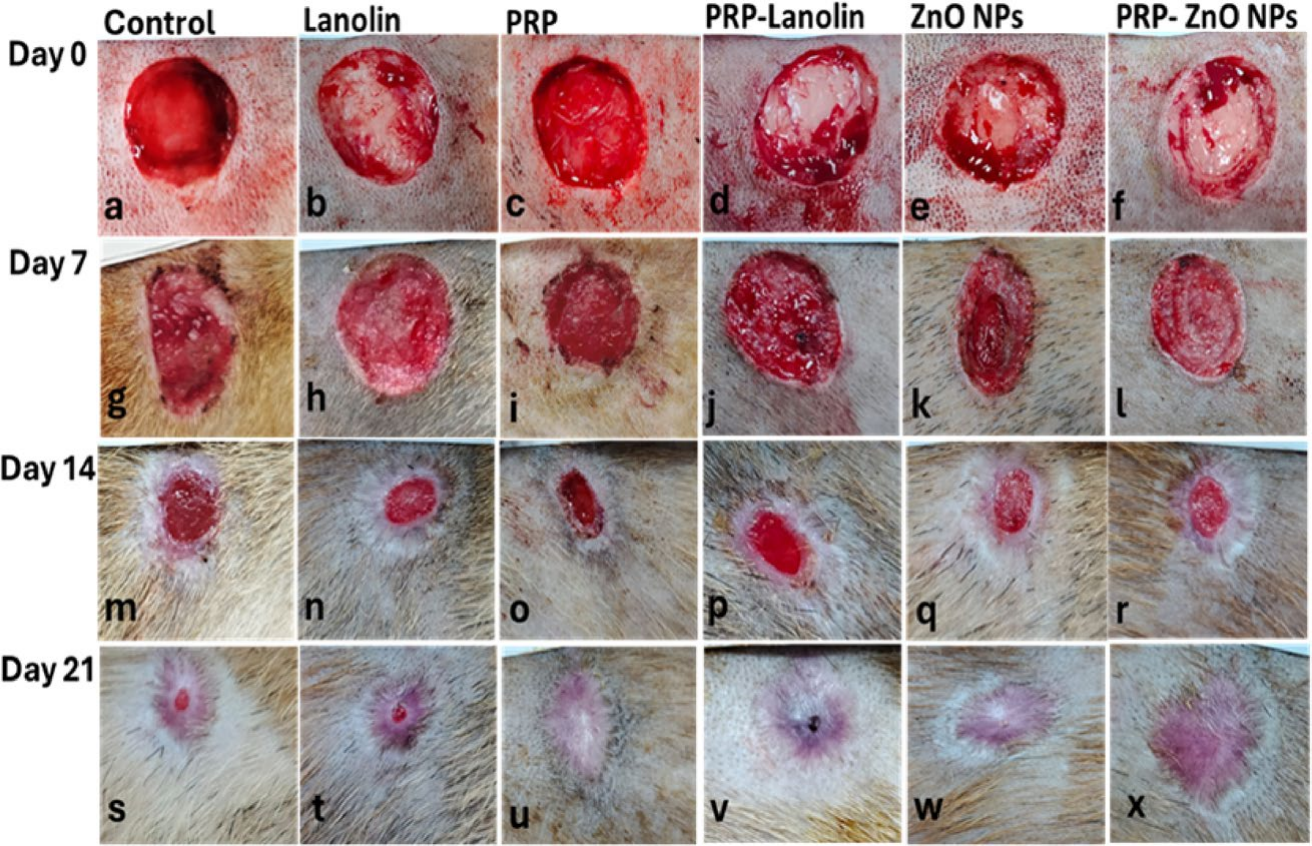
Representative photographs of wound healing in all groups at days 0 **(a-f)**, 7 **(g-i)**, 14 **(m-r)** and 21 **(s-x)**.

The wounds at day 0 were similar in size in all groups **(**Figs. 4a-f**)**. After 7 and 14 days, groups 3 (PRP-treated wound), 5 (ZnO NPs treated wounds), and 6 (PRP-ZnO NPs treated wounds) showed smaller sized wounds in comparison to groups 1 (control), 2 (lanolin treated wounds) and 4 (PRP-lanolin treated wounds) as shown in Figs. 4g-r. After 21 days, wounds in groups 3, 5, and 6 showed complete healing and wound closure, unlike other groups **(**Fig. 4s-x**)**.

### Palnimetry findings

#### Wound size percent

There was a significant interaction effect of the group and time for wound size percent (*P* < 0.001). Pairwise comparisons for wound size percent between the four-time intervals of assessment revealed significant differences between all-time intervals (*P* < 0.001). All groups began the study with comparable wound sizes percent (100) on day 0, with no significant differences detected (*P* not computed).

By day 7, a significant reduction in wound size% was observed across all groups (*P* ≤ 0.001). The PRP-treated group exhibited the most pronounced reduction in wound size (68.18 ± 9.77), followed by ZnO NPs (72.93 ± 3.79) and PRP–ZnO NPs (73.66 ± 4.41) groups. In contrast, the control (83.39 ± 7.08), lanolin (88.17 ± 7.08) and PRP-lanolin (81.13 ± 9.51) groups showed the least improvement, maintaining large wound sizes.

At day 14, wound size% reduction remained significantly more pronounced in the PRP–ZnO NPs (25.91 ± 3.11) than PRP groups (28.77 ± 2.31), ZnO NPs (30.5 ± 2.55), PRP–lanolin (32.77 ± 3.36) and lanolin (35.44 ± 4.54) groups which were also demonstrated better outcomes than control group (*P* ≤ 0.001), but were less effective than PRP–ZnO NPs.

**Fig. 5:**
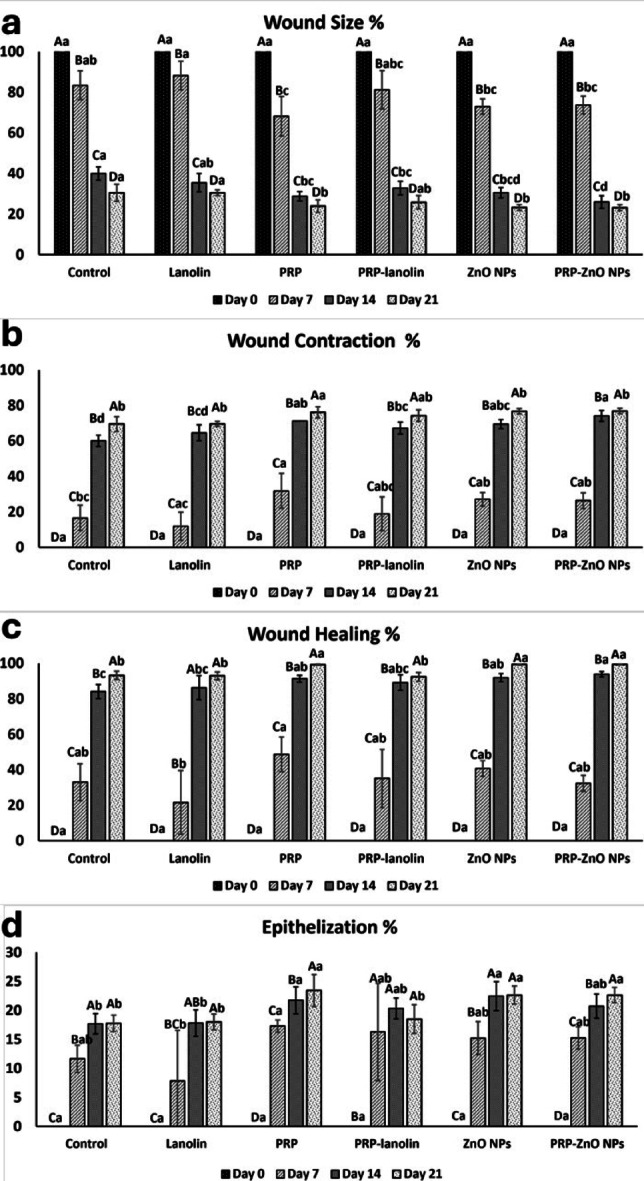
Clustered bar graph (mean, SD) representing planimetric evaluation as determined by wound size %, wound contraction %, wound healing % and epithelialization % evaluated in different groups at days 0, 7, 14, and 21. Different upper-case letters within the same group indicate statistically significant differences. Different lower-case letters within the same time point indicate statistically significant difference (*P* < 0.001).

By day 21, the smallest wound sizes were recorded in the PRP–ZnO NPs (23.17 ± 1.47), ZnO NPs (23.22 ± 1.46), and PRP (23.92 ± 3.12) groups, which were significantly smaller than those of the control and lanolin groups (*P* ≤ 0.001). The PRP–lanolin (25.82 ± 3.26) group also showed improved healing compared to control and lanolin groups but remained less effective than the PRP–ZnO NPs group **(**Fig. 5**)**.

In summary, wound healing progressed in all groups over time, with the PRP–ZnO NPs combination consistently producing the most substantial wound size reduction, followed closely by PRP and ZnO NPs alone. The control and lanolin groups were the least effective throughout the study period as shown in Table 2; Fig. 5.

**Table 2:** Descriptive statistics for wound size%, wound contraction%, wound healing% and epithelialization% in all groups at days 0, 7, 14, and 21.

Variables	Groups	Day 0	Day 7	Day 14	Day 21	*P*- value
**Wound size percent**	Control	100^A a^	83.39 ± 7.08^Bab^	39.98 ± 3.21^Ca^	30.51 ± 4.24^Da^	≤ 0.001 *
	Lanolin	100^A a^	88.17 ± 7.08^Ba^	35.44 ± 4.54^Cab^	30.46 ± 1.34^Da^	≤ 0.001 *
	PRP	100 ^Aa^	68.18 ± 9.77^Bc^	28.77 ± 2.31^Cbc^	23.92 ± 3.12^Db^	≤ 0.001 *
	PRP-lanolin	100^Aa^	81.13 ± 9.51^Babc^	32.77 ± 3.36^Cbc^	25.82 ± 3.26^Dab^	≤ 0.001 *
	ZnO NPs	100 ^Aa^	72.93 ± 3.79^Bbc^	30.5 ± 2.55^Cbcd^	23.22 ± 1.46^Db^	≤ 0.001 *
	PRP-ZnO NPs	100 ^Aa^	73.66 ± 4.41^Bbc^	25.91 ± 3.11^Cd^	23.17 ± 1.47^Db^	≤ 0.001 *
	***P- value***	Not computed	≤ 0.001 *	≤ 0.001 *	≤ 0.001 *	
**Wound contraction percent**	Control	0.00^Da^	16.61 ± 7.08^Cbc^	60.02 ± 3.21^Bd^	69.49 ± 4.24^Ab^	≤ 0.001 *
	Lanolin	0.00^Da^	11.83 ± 7.87^Cc^	64.55 ± 4.45^Bcd^	69.54 ± 1.35^Ab^	≤ 0.001 *
	PRP	0.00^Da^	31.83 ± 9.77^Ca^	71.23 ± 2.31^Bab^	76.08 ± 3.12^Aa^	≤ 0.001 *
	PRP-lanolin	0.00^Da^	18.87 ± 9.51^Cabc^	67.23 ± 3.36^Bbc^	74.18 ± 3.26^Aab^	≤ 0.001 *
	ZnO NPs	0.00^Da^	27.07 ± 3.79^Cab^	69.5 ± 2.55^Babc^	76.78 ± 1.46^Aa^	≤ 0.001 *
	PRP-ZnO NPs	0.00^Da^	26.34 ± 4.4^Cab^	74.09 ± 3.11^Ba^	76.83 ± 1.47^Aa^	≤ 0.001 *
	***P*** **- value**	Not computed	≤ 0.001 *	≤ 0.001 *	≤ 0.001 *	
**Healing percent**	Control	0.00^Da^	33.05 ± 10.42^Cab^	84.16 ± 4.04^Bc^	93.2 ± 2.36^Ab^	≤ 0.001 *
	Lanolin	0.00^Ca^	21.63 ± 17.94^Bb^	86.34 ± 6.77^Abc^	92.98 ± 2.22^Ab^	≤ 0.001 *
	PRP	0.00^Da^	48.7 ± 9.77^Ca^	91.38 ± 1.9^Bab^	99.31 ± 0.38^Aa^	≤ 0.001 *
	PRP-lanolin	0.00^Da^	35.16 ± 16.36^Cab^	89.18 ± 4.31^Babc^	92.44 ± 2.5^Ab^	≤ 0.001 *
	ZnO NPs	0.00^Da^	40.76 ± 4.34^Cab^	91.95 ± 2.29^Bab^	99.44 ± 0.41^Aa^	≤ 0.001 *
	PRP-ZnO NPs	0.00^Da^	32.39 ± 4.45^Cab^	93.9 ± 1.46^Ba^	99.46 ± 0.32^Aa^	≤ 0.001 *
	***P*** **- value**	Not computed	0.012*	0.001 *	≤ 0.001 *	
**Epithelialization percent**	Control	0.00^Ca^	11.67 ± 2.33^Bab^	17.72 ± 1.75^Ab^	17.78 ± 1.4^Ab^	≤ 0.001 *
	Lanolin	0.00^Ca^	7.85 ± 8.72^BCb^	17.81 ± 2.3^ABb^	18.01 ± 1.36^Ab^	≤ 0.001 *
	PRP	0.00^Da^	17.31 ± 1.06^Ca^	21.75 ± 2.32^Ba^	23.43 ± 2.76^Aa^	≤ 0.001 *
	PRP-lanolin	0.00^Ba^	16.29 ± 8.41^Aab^	20.35 ± 1.79^Aab^	18.5 ± 2.47^Ab^	≤ 0.001 *
	ZnO NPs	0.00^Ca^	15.23 ± 2.83^Bab^	22.46 ± 2.5^Aa^	22.65 ± 1.55^Aa^	≤ 0.001 *
	PRP-ZnO NPs	0.00^Da^	15.25 ± 1.93^Cab^	20.75 ± 2.08^Bab^	22.63 ± 1.33^Aa^	≤ 0.001 *
	***P*** **- value**	Not computed	0.038*	0.002*	≤ 0.001 *	

Means with different capital letters in the same row indicate significant difference.

Means with different small letters in the same column indicate significant difference. *Significant at *P* < 0.05.

#### Wound contraction percent

There was a significant interaction effect of group and time for wound contraction percent (*P* < 0.001). Pairwise comparisons for wound contraction percent between the four-time intervals of the assessment revealed significant differences between all-time intervals (*P* < 0.001). At day 0, all groups exhibited 0% wound contraction as expected, with no significant differences among them. By day 7, a significant increase in wound contraction was observed in all treated groups. The PRP group showed the highest contraction percentage (31.83 ± 9.77), followed by ZnO NPs (27.07 ± 3.79%) and PRP–ZnO NPs (26.34 ± 4.4). These values were significantly greater than those recorded in lanolin (11.83 ± 7.87), and PRP treated group was significantly different from the control (16.61 ± 7.08) and indicating enhanced early healing in PRP and nanoparticle-treated wounds (*P* ≤ 0.001). PRP-lanolin group showed moderate wound contraction than other effective groups (18.87 ± 9.51).

By day 14, wound contraction% further progressed in all groups (*P* ≤ 0.001). The PRP–ZnO NPs group showed the greatest contraction (74.09 ± 3.11), followed closely by PRP (71.23 ± 2.31) and ZnO NPs (69.5 ± 2.55), all of which were significantly higher than the control (60.02 ± 3.21) and lanolin (64.55 ± 4.45) groups. The PRP–Lanolin group (67.23 ± 3.36) also demonstrated marked improvement over the control group.

At day 21, wound contraction% reached its highest levels across all treatment groups. The PRP–ZnO NPs (76.83 ± 1.47), ZnO NPs (76.78 ± 1.46) and PRP (76.08 ± 3.12%) groups achieved the greatest contraction percentages, significantly outperforming the control (69.49 ± 4.24) and lanolin (69.54 ± 1.35) groups (*P* ≤ 0.001). PRP–Lanolin (74.18 ± 3.26) also showed superior results compared to the control and lanolin groups but not significantly different (*P* > 0.05) as shown in Table 2; Fig. 5.

In conclusion, PRP, ZnO NPs, and their combination significantly enhanced wound contraction compared to the control and lanolin. The PRP–ZnO NPs group showed the most pronounced effect, indicating a synergistic role in accelerating wound closure.

#### Healing percent

There was a significant interaction effect of the group and time for healing percent (*P* < 0.001). Pairwise comparisons for healing percent between the four-time intervals of the assessment revealed significant differences between all-time intervals (*P* < 0.001). At day 0, all groups showed 0% healing, with no significant differences observed among them.

By day 7, the PRP group exhibited the highest healing percent (48.7 ± 9.77), which was significantly greater than lanolin (21.63 ± 17.94). Following that the ZnO NPs (40.76 ± 4.34) and PRP–Lanolin (35.16 ± 16.36), control (33.05 ± 10.42), and PRP–ZnO NPs (32.39 ± 4.45), and these groups showed moderate improvement with no significant difference between them (*P* > 0.05).

At day 14, all treatment groups exhibited significant enhancement in healing percentage compared to the control (*P* = 0.001) except lanolin and PRP-lanolin groups. PRP–ZnO NPs (93.9 ± 1.46), ZnO NPs (91.95 ± 2.29), and PRP (91.38 ± 1.9) achieved the highest healing levels and were significantly better than the control (84.16 ± 4.04). PRP–Lanolin (89.18 ± 4.31) and lanolin (86.34 ± 6.77) showed intermediate results.

By day 21, maximum healing was observed in all treatment groups. PRP–ZnO NPs (99.46 ± 0.32), ZnO NPs (99.44 ± 0.41), and PRP (99.31 ± 0.38) achieved nearly complete healing and were statistically superior to the control (93.2 ± 2.36), lanolin (92.98 ± 2.22) and PRP-lanolin (92.44 ± 2.5) where *P* < 0.001.

In conclusion, PRP-ZnO NPs, ZnO NPs, and PRP, significantly enhanced wound healing compared to control and lanolin as shown in Table 2; Fig. 5.

#### Epithelialization percent

There was a significant interaction effect of the group and time for epithelialization percent (*P* < 0.001). Pairwise comparisons for epithelization percent between the four-time intervals of the assessment revealed significant differences between all-time intervals (*P* < 0.001) except between the third- and fourth-time intervals. At day 0, all groups recorded 0% epithelialization with no significant differences (*P* not computed).

By day 7, PRP (17.31 ± 1.06) group showed significantly greater epithelialization% compared to the lanolin group (7.85 ± 8.72) (*P* = 0.038). PRP–Lanolin (16.29 ± 8.41), ZnO NPs (15.23 ± 2.83), PRP–ZnO NPs (15.25 ± 1.93) and control (11.67 ± 2.33) also demonstrated improved outcomes with no significant difference between them (*P* > 0.05).

At day 14, epithelialization progressed significantly in all treated groups (*P* = 0.002). The highest values were observed in ZnO NPs (22.46 ± 2.5), PRP (21.75 ± 2.32), which were significantly greater than those of the control (17.72 ± 1.75) and Lanolin (17.81 ± 2.3) groups. Following that PRP–ZnO NPs (20.75 ± 2.08), and PRP-lanolin (20.35 ± 1.79) with no significant difference between them and all groups (*P* > 0.05).

By day 21, all treated groups showed advanced epithelialization, ZnO NPs (22.65 ± 1.55), PRP–ZnO NPs (22.63 ± 1.33), and PRP (23.43 ± 2.76) achieved the highest epithelialization levels significantly different from PRP–lanolin group (18.5 ± 2.47), lanolin (18.01 ± 1.36), and control (17.78 ± 1.4) (*P* < 0.001), as shown in Table 2; Fig. 5.

In conclusion, PRP-ZnO NPs, ZnO NPs, and PRP, significantly enhanced wound epithelization compared to other groups.

### Findings of the biochemical markers

#### Total Antioxidant Capacity (TAC)

There was a significant interaction effect of the group and time for TAC (F = 5.736, *P* < 0.001). There was a significant main effect of time for TAC (F = 279.712, *P* < 0.001). At day 0, TAC values were statistically comparable among all groups (*P* = 0.098), with no significant differences observed.

By day 5, significant changes were detected (*P* ≤ 0.001), with the control group showing a marked increase in TAC (2.09 ± 0.11 mM/L), while all other treatment groups especially PRP (0.52 ± 0.02 mM/L), PRP–lanolin (0.9 ± 0.15 mM/L), ZnO NPs (0.93 ± 0.06 mM/L), and PRP–ZnO NPs (1.03 ± 0.4 mM/L) exhibited significantly lower values (*P* < 0.05), indicating effective antioxidant consumption during active wound healing.

At day 10, TAC levels declined significantly in all groups (*P* ≤ 0.001). The lowest levels were observed in the PRP (0.21 ± 0.01 mM/L) and the control (0.27 ± 0.01 mM/L) groups which were significantly lower than ZnO NPs (0.66 ± 0.51 mM/L), PRP–ZnO NPs (0.81 ± 0.03 mM/L) and PRP–lanolin (0.85 ± 0.15 mM/L) groups (*P* < 0.05). While lanolin group (1.54 ± 0.08 mM/L) maintained relatively higher value, suggesting slower oxidative turnover.

**Fig. 6:**
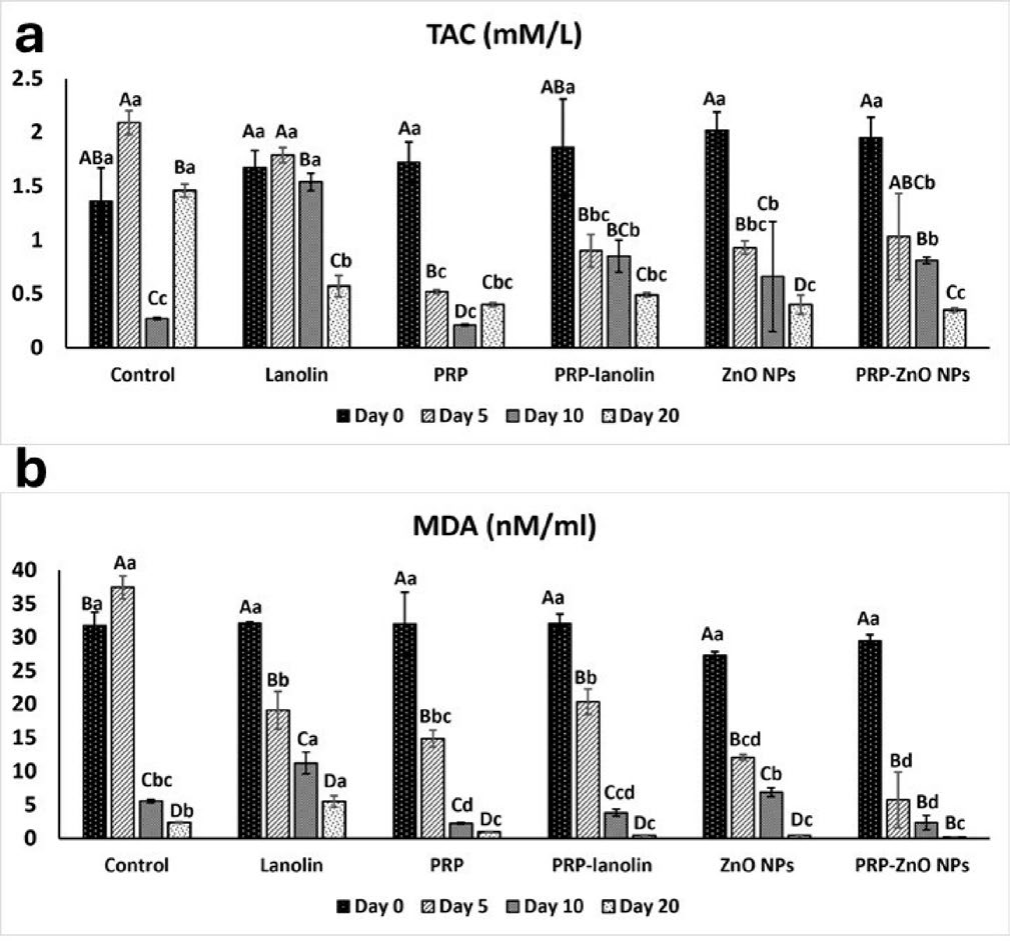
Clustered bar graph (mean, SD) representing total antioxidant capacity (TAC) and malondialdehyde (MDA) evaluated in different groups at days 0, 5, 10, and 20. Different upper-case letters within the same group indicate statistically significant differences. Different lower-case letters within the same time point indicate statistically significant difference (*P* < 0.001).

By day 20, PRP–ZnO NPs (0.35 ± 0.02 mM/L), ZnO NPs (0.4 ± 0.09 mM/L) were significantly lower than lanolin (0.57 ± 0.1 mM/L) and control (1.46 ± 0.06 mM/L) groups (*P* ≤ 0.001). Following that, PRP-lanolin (0.49 ± 0.02 mM/L) and PRP (0.4 ± 0.02) had significantly lower values than those of the control (1.46 ± 0.06 mM/L) group (*P* ≤ 0.001). This persistent decrease reflected elevated antioxidant utilization associated with enhanced tissue repair activity, as shown in Table 3; Fig. 6.

**Table 3:** Descriptive statistics for biochemical markers in all groups at days 0, 5, 10, and 20.

Variables	Groups	Day 0	Day 5	Day 10	Day 20	*P*- value
**TAC (mM/L)**	Control	1.36 ± 0.31 ^AB, a^	2.09 ± 0.11 ^A, a^	0.27 ± 0.01 ^C, c^	1.46 ± 0.06 ^B, a^	≤ 0.001*
	Lanolin	1.67 ± 0.16 ^A,.a^	1.79 ± 0.07 ^A, a^	1.54 ± 0.08 ^B, a^	0.57 ± 0.1 ^C, b^	≤ 0.001*
	PRP	1.72 ± 0.19 ^A, a^	0.52 ± 0.02 ^B, c^	0.21 ± 0.01 ^D, c^	0.4 ± 0.02 ^C, bc^	≤ 0.001*
	PRP-Lanolin	1.86 ± 0.45 ^AB, a^	0.9 ± 0.15 ^B, bc^	0.85 ± 0.15 ^BC, b^	0.49 ± 0.02 ^C, bc^	0.004*
	ZnO NPs	2.02 ± 0.17 ^A, a^	0.93 ± 0.06 ^B, bc^	0.66 ± 0.51^C, b^	0.4 ± 0.09 ^D, c^	≤ 0.001*
	PRP-ZnO NPs	1.95 ± 0.19 ^A, a^	1.03 ± 0.4 ^ABC, b^	0.81 ± 0.03 ^B, b^	0.35 ± 0.02 ^C, c^	0.001*
	***P*** **- value**	0.098	≤ 0.001*	≤ 0.001*	≤ 0.001*	
**MDA (nM/ml)**	Control	31.76 ± 1.93 ^B, a^	37.42 ± 1.69 ^A, a^	5.57 ± 0.26 ^C, bc^	2.37 ± 0.11 ^D b^	≤ 0.001*
	Lanolin	32.1 ± 0.13 ^A, a^	19.1 ± 2.81 ^B, b^	11.24 ± 1.61 ^C, a^	5.51 ± 0.86 ^D, a^	≤ 0.001*
	PRP	32 ± 4.68 ^A, a^	14.87 ± 1.26 ^B, bc^	2.25 ± 0.15 ^C, d^	0.94 ± 0.06 ^D, c^	≤ 0.001*
	PRP-Lanolin	32.06 ± 1.39 ^A, a^	20.4 ± 1.91 ^B, b^	3.83 ± 0.52 ^C, cd^	0.42 ± 0.05 ^D, c^	≤ 0.001*
	ZnO NPs	27.25 ± 0.61 ^A, a^	12.07 ± 0.45 ^B, cd^	6.87 ± 0.64 ^C, b^	0.45 ± 0.05 ^D, c^	≤ 0.001*
	PRP-ZnO NPs	29.43 ± 0.95 ^A, a^	5.73 ± 4.13 ^B, d^	2.35 ± 1.1 ^B, d^	0.14 ± 0.02 ^B, c^	≤ 0.001*
	***P*** **- value**	0.088	≤ 0.001*	≤ 0.001*	≤ 0.001*	
**MEPE**	Control	1 ^Ba^	1.49 ± 0.036^Ac^	0.31 ± 0.02^Cb^	0.14 ± 0.02^Dd^	≤ 0.001*
	Lanolin	1 ^ABa^	1.46 ± 0.21^Ac^	0.87 ± 0.19^Bb^	0.1 ± 0.02^Cd^	≤ 0.001*
	PRP	1 ^Ca^	13.43 ± 1.75^Ab^	1.5 ± 0.58^Cb^	4.13 ± 0.3^Bb^	≤ 0.001*
	PRP-lanolin	1 ^Ca^	1.63 ± 0.18^ABc^	1.49 ± 0.03^Ab^	1.15 ± 0.14^Cc^	0.002
	ZnO NPs	1 ^Aa^	0.18 ± 0.03^Dc^	0.3 ± 0.02^Cb^	0.37 ± 0.03^Bd^	≤ 0.001*
	PRP-ZnO NPs	1 ^Da^	33.64 ± 1.44^Aa^	23.2 ± 0.9^Ba^	12.99 ± 0.09^Ca^	≤ 0.001*
	***P*** **- value**	Not computed	≤ 0.001*	≤ 0.001*	≤ 0.001*	
**TGF β**	Control	1 ^Ba^	1.85 ± 0.03^Ab^	0.49 ± 0.19^Cc^	0.3 ± 0.18^Dc^	≤ 0.001*
	Lanolin	1 ^Aa^	0.08 ± 0.01^Cb^	0.09 ± 0.01^Bc^	0.16 ± 0.07^BCc^	≤ 0.001*
	PRP	1 ^Da^	8.47 ± 0.13^Ba^	9.6 ± 0.21^Aa^	7.11 ± 0.29^Ca^	≤ 0.001*
	PRP-lanolin	1 ^Ca^	1.24 ± 0.08^Bb^	1.54 ± 0.03^Ab^	0.1 ± 0.02^Dc^	≤ 0.001*
	ZnO NPs	1 ^Aa^	0.21 ± 0.01^Cb^	0.17 ± 0.01^BCc^	0.52 ± 0.15^Bc^	≤ 0.001*
	PRP-ZnO NPs	1 ^BCa^	8.75 ± 2.28^Aa^	2.19 ± 0.6^ABb^	2.72 ± 1.1^ABb^	0.003*
	***P*** **- value**	Not computed	≤ 0.001*	≤ 0.001*	≤ 0.001*	
**TNF α**	Control	1 ^Ca^	0.49 ± 0.07^Dc^	18.1 ± 0.18^Aa^	6.16 ± 0.22^Ba^	≤ 0.001*
	Lanolin	1 ^Ca^	1.53 ± 0.05^Ba^	1.8 ± 0.04^Ab^	2.35 ± 0.3^Ab^	≤ 0.001*
	PRP	1 ^Aa^	0.59 ± 0.03^Bc^	0.6 ± 0.08^Bc^	0.13 ± 0.02^Cd^	≤ 0.001*
	PRP-lanolin	1 ^Aa^	1 ± 0.05^Ab^	0.1 ± 0.01^Cd^	0.25 ± 0.02^Bd^	≤ 0.001*
	ZnO NPs	1 ^Aa^	0.21 ± 0.16^BCc^	0.18 ± 0.02^Cd^	0.43 ± 0.04^Bd^	≤ 0.001*
	PRP-ZnO NPs	1 ^Aa^	1.2 ± 0.3^Aab^	0.9 ± 0.3^Ac^	1.27 ± 0.45^Ac^	0.588
	***P*** **- value**	Not computed	≤ 0.001*	≤ 0.001*	≤ 0.001*	
**PDGFβ**	Control	13.2 ± 1.55^Ca^	36.82 ± 2.17^Ab^	27.87 ± 1.37^Bd^	12.52 ± 1.26^Cc^	≤ 0.001 *
	Lanolin	15.23 ± 2.15^Ca^	33.27 ± 2.1^Ab^	28.49 ± 0.84^Bd^	14.27 ± 1.26^Cc^	≤ 0.001 *
	PRP	15.23 ± 2.9^Da^	81.5 ± 6.5^Ca^	129.67 ± 4.51^Ba^	167.87 ± 8.63^Aa^	≤ 0.001 *
	PRP -lanolin	14.37 ± 0.78^Da^	72.03 ± 5.58^Ca^	104.4 ± 5.92^Bc^	137.17 ± 3.69^Ab^	≤ 0.001 *
	ZnO NPs	15.8 ± 1.73^Ba^	39.07 ± 3.69^Ab^	33.07 ± 3.72^Ad^	15.6 ± 2.31^Bc^	≤ 0.001 *
	PRP -ZnO NPs	12.33 ± 1.61^Da^	77.07 ± 3.41^Ca^	118.57 ± 3.37^Bb^	162.53 ± 12.12^Aa^	≤ 0.001 *
	***P*** **- value**	0.268	≤ 0.001*	≤ 0.001*	≤ 0.001*	

Means with different capital letters in the same row indicate significant difference. Means with different small letters in the same column indicate significant difference. *Significant at *P* < 0.05.

TAC levels decreased significantly over time in all treated groups, with PRP, ZnO NPs, and PRP–ZnO NPs showing the most pronounced decline, reflecting high antioxidant activity during accelerated healing.

#### Malondialdehyde (MDA)

There was a significant interaction effect of the group and time for MDA (F = 5.902, *P* < 0.001). There was a significant main effect of time for MDA (F = 1075.971, *P* < 0.001). At day 0, MDA levels were comparable across all groups, with no statistically significant differences observed (*P* = 0.088).

By day 5, a significant reduction in MDA concentrations was recorded in the PRP–ZnO NPs group (5.73 ± 4.13 nM/ml) which was significantly different from all groups except ZnO NPs group (12.07 ± 0.45), following that PRP (14.87 ± 1.26), lanolin (19.1 ± 2.81), and PRP-lanolin (20.4 ± 1.91) groups which were significantly lower than the control group (37.42 ± 1.69) where *P* ≤ 0.001.

At day 10, the lowest MDA levels indicating effective reduction of oxidative stress were seen in the PRP (2.25 ± 0.15) and PRP–ZnO NPs (2.35 ± 1.1 nM/ml) groups which were significantly lower than those of all groups except PRP-lanolin (3.83 ± 0.52), following that control (5.57 ± 0.26) and ZnO NPs (6.87 ± 0.64), which were significantly lower than the lanolin group (11.24 ± 1.61) that remaining the highest value (*P* ≤ 0.001).

By day 20, the most pronounced antioxidant effect was recorded in the PRP–ZnO NPs group (0.14 ± 0.02 nM/ml), PRP–lanolin (0.42 ± 0.05) and ZnO NPs (0.45 ± 0.05), and PRP (0.94 ± 0.06) groups which had significantly lower MDA levels than lanolin (5.51 ± 0.86), and control (2.37 ± 0.11) groups (*P* ≤ 0.001), as shown in Table 3; Fig. 6.

PRP–ZnO NPs markedly reduced MDA levels over time, indicating strong antioxidant activity. PRP, ZnO NPs, and PRP–lanolin also showed effective suppression of lipid peroxidation compared to the control and lanolin groups.

### Findings of the gene expression

#### Matrix extracellular phosphoglycoprotein (MEPE)

There was a significant interaction effect of the group and time for MEPE (F = 23.572, *P* < 0.001). There was a significant main effect of time for MEPE (F = 1725.127, *P* < 0.001). At day 0, no significant differences in MEPE levels were found among all groups (*P* not computed).

By day 5, a significant increase was observed in the PRP–ZnO NPs group (33.64 ± 1.44), which was markedly higher than all other groups (*P* ≤ 0.001). PRP alone (13.43 ± 1.75) also showed significantly higher MEPE expression than PRP–lanolin (1.63 ± 0.18), ZnO NPs (0.18 ± 0.03), lanolin (1.46 ± 0.21), and control (1.49 ± 0.036) groups.

At day 10, the PRP–ZnO NPs group maintained the significantly higher MEPE level (23.2 ± 0.90) than PRP (1.5 ± 0.58), PRP–lanolin (1.49 ± 0.03), lanolin (0.87 ± 0.19), control (0.31 ± 0.02), and ZnO NPs (0.3 ± 0.02) groups (*P* ≤ 0.001).

**Fig. 7:**
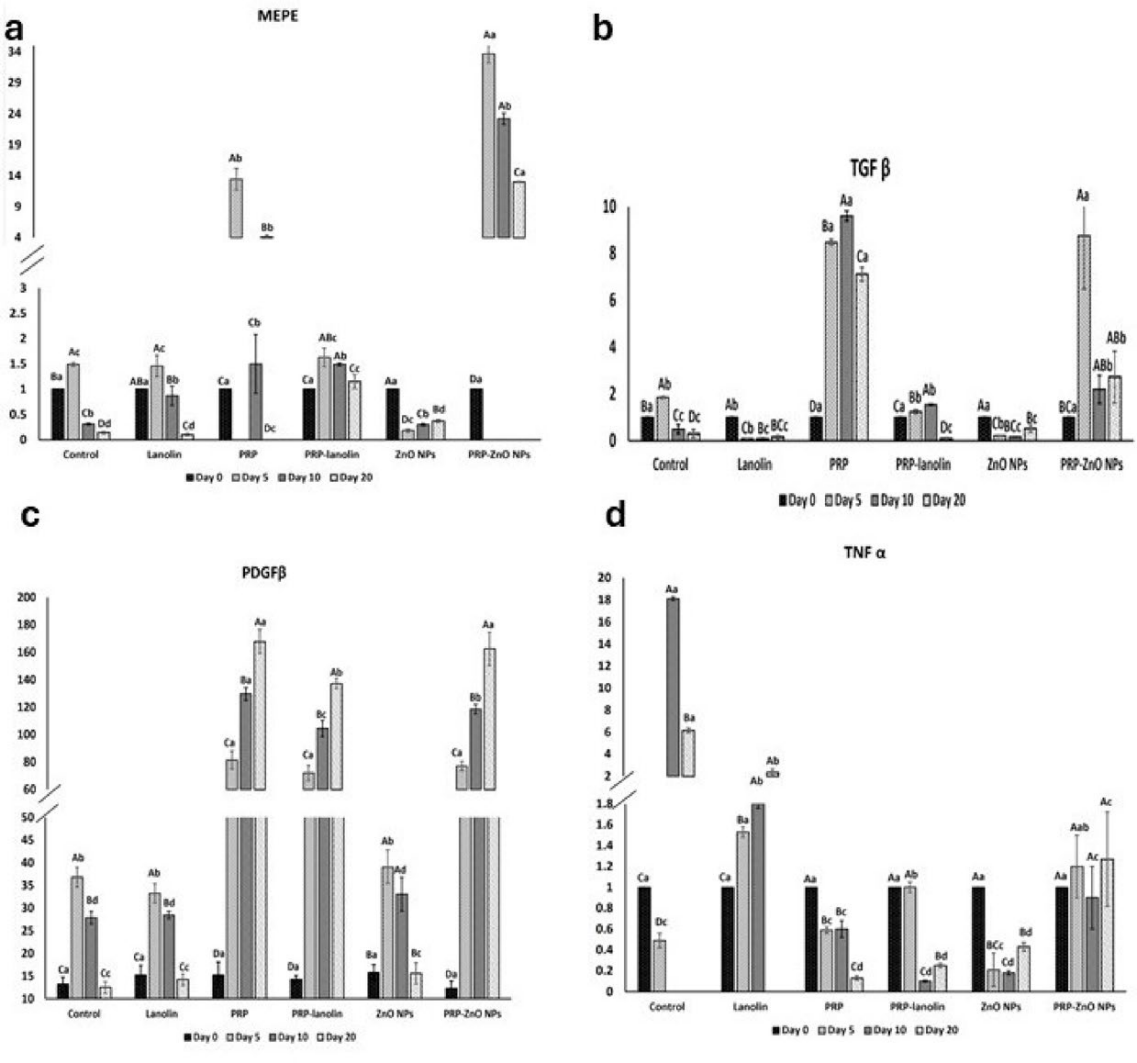
Clustered bar graph (mean, SD) representing gene expression of matrix extracellular phosphoglycoprotien (MEPE), transforming growth factor beta (TGF-β) and tumor necrosis factor alpha (TNF-α) and the concentration of platelets derived growth factor beta (PDGFβ) evaluated in different groups at days 0, 5, 10, and 20. Different upper-case letters within the same group indicate statistically significant differences. Different lower-case letters within the same time point indicate statistically significant difference (*P* < 0.001).

By day 20, the PRP–ZnO NPs group continued to exhibit the greatest MEPE expression (12.99 ± 0.09), with PRP (4.13 ± 0.3) and PRP–lanolin (1.15 ± 0.14), also demonstrating significantly elevated levels compared to the control (0.14 ± 0.02), lanolin (0.1 ± 0.02), and ZnO NPs (0.37 ± 0.03) groups (*P* ≤ 0.001), as shown in Table 3; Fig. 7.

A strong time-dependent increase in MEPE was noted particularly in the PRP–ZnO NPs group, followed by PRP and PRP–lanolin groups, reflecting enhanced tissue regeneration capacity.

#### Transforming growth factor beta (TGF-β)

There was a significant interaction effect of the group and time for TGF-β (F = 12.047, *P* < 0.001). There was a significant main effect of time for TGF-β (F = 1018.467, *P* < 0.001). A statistically significant difference (*P* ≤ 0.001) in TGF-β levels was observed among the groups and overtime. At baseline, all groups showed equal and minimal TGF-β levels (1 pg/mL), with no statistically significant differences (*P* > 0.05). This confirms a homogeneous starting point before treatment.

At day 5, PRP–ZnO NPs (8.75 ± 2.28) and PRP (8.47 ± 0.13) groups recorded the significantly greater TGF-β expression (*P* ≤ 0.001) than all other groups, control (1.85 ± 0.03) and PRP–lanolin (1.24 ± 0.08) groups which exhibited moderate increases. ZnO NPs (0.21 ± 0.01) and lanolin (0.08 ± 0.01) groups remained significantly lower.

At day 10, PRP continued to exhibit the significantly highest TGF-β level (9.60 ± 0.21) among all groups. PRP–ZnO NPs (2.19 ± 0.6) and PRP–lanolin (1.54 ± 0.03) groups showed a moderate increase that was significantly higher than those of the control (0.49 ± 0.19), ZnO NPs (0.17 ± 0.01) and lanolin (0.09 ± 0.01) groups (*P* ≤ 0.001).

At day 20, PRP (7.11 ± 0.29) maintained the significantly highest sustained TGF-β expression among all groups. PRP–ZnO NPs (2.72 ± 1.1) was the second highest and significantly higher than ZnO NPs (0.52 ± 0.15), control (0.30 ± 0.18), lanolin (0.16 ± 0.07) and PRP–lanolin (0.10 ± 0.01) groups (*P* ≤ 0.001), as shown in Table 3; Fig. 7.

At all post-treatment time points, PRP demonstrated the highest and most consistent increase in TGF-β levels, indicating a superior role in stimulating fibroblast activation, extracellular matrix deposition, and wound remodeling. PRP–ZnO NPs showed moderate activity with delayed peak. In contrast, lanolin and ZnO NPs alone had minimal effects, confirming that platelet-derived factors are the primary contributors to TGF-β upregulation in this model.

#### Tumor necrosis factor-alpha (TNF-α)

There was a significant interaction effect of the group and time for TNF***-α*** (F = 12.710, *P* = 0.003). There was a significant main effect of time for TNF***-α*** (F = 13370.908, *P* < 0.001). At Day 0, all groups started with identical values (1), indicating no significant differences in TNF-α levels among groups at baseline, (*P-*value not computed).

At Day 5, the lanolin group showed the highest TNF-α level (1.53 ± 0.05), which was significantly higher than all other groups except PRP-ZnO NPs (1.2 ± 0.3). Following that, PRP-lanolin (1 ± 0.05) group was significantly higher than all except PRP-ZnO NPs (*P* ≤ 0.001). The lowest levels of TNF-α were reported in PRP (0.59 ± 0.03), control (0.49 ± 0.07) and ZnO NPs (0.21 ± 0.16) groups, with no significant differences between them (*P* > 0.05).

At Day 10, the control (18.1 ± 0.18) and lanolin (1.8 ± 0.04) groups exhibited significantly higher TNF-α levels than all treated groups (*P* ≤ 0.001). The PRP-ZnO NPs (0.9 ± 0.3) and PRP (0.6 ± 0.08) groups were significantly higher than PRP-lanolin (0.1 ± 0.01) and ZnO NPs (0.18 ± 0.02) groups (*P* ≤ 0.001).

At Day 20, the control (6.16 ± 0.22) and lanolin (2.35 ± 0.3) groups remained significantly higher than all other groups. The PRP-ZnO NPs group (1.27 ± 0.45) was significantly higher than PRP (0.13 ± 0.02), PRP-lanolin (0.25 ± 0.02), and ZnO NPs (0.43 ± 0.04) groups (*P* ≤ 0.001), which showed no significant differences among each other, as shown in Table 3; Fig. 7.

Across all groups, TNF-α levels showed significant temporal reductions from Day 5 to Day 21. This pattern was particularly evident in the treated groups, reflecting an improved anti-inflammatory response over time.

Although all treatments led to a reduction in TNF-α levels over time, PRP alone was the most effective in lowering TNF-α, especially by Day 20. ZnO NPs also showed strong anti-inflammatory effects. PRP-ZnO NPs, however, had less favorable anti-inflammatory outcomes than PRP or ZnO NPs alone.

#### Platelets derived growth factor beta PDGFβ

There was a significant interaction effect of the group and time for PDGF**β** (*P* = 0.003). There was a significant main effect of time for PDGF**β** between the four-time intervals of assessment (*P* < 0.001). At day 0, there were no statistically significant differences among groups (*P* = 0.268), and the PDGF**β** levels were similar across all groups **(**Table 3**).**

At day 7, a significant difference was observed between PRP group that showed the highest PDGF**β** level (81.5 ± 6.5), followed by PRP–ZnO NPs (77.07 ± 3.41) and PRP–lanolin (72.03 ± 5.58) groups. The control (36.82 ± 2.17), lanolin (33.27 ± 2.1), and ZnO NPs (39.07 ± 3.69) groups had comparable and significantly lower values than other groups (*P* ≤ 0.001).

At day 14, PRP (129.67 ± 4.51) and PRP–ZnO NPs (118.57 ± 3.37) groups showed significantly higher PDGF**β** levels compared to all other groups. PRP–lanolin (104.4 ± 5.92) followed, with intermediate levels, and all were significantly higher than lanolin (28.49 ± 0.84), control (27.87 ± 1.37), and ZnO NPs (33.07 ± 3.72) groups (*P* ≤ 0.05).

At day 21, PRP group had the highest PDGF**β** level (167.87 ± 8.63), closely followed by PRP–ZnO NPs group (162.53 ± 9.91), and both were significantly higher than PRP–lanolin (137.17 ± 3.69). All three PRP-containing groups were significantly superior to the ZnO NPs (15.6 ± 2.31), lanolin (14.27 ± 1.26), and control (12.52 ± 1.26) groups, which maintained the lowest PDGF**β** expression **(**Table 3**)**.

The PDGF**β** level significantly increased over time in all groups, with the most pronounced and sustained elevation observed in the PRP and PRP–ZnO NP groups. These findings highlighted the potent stimulatory effect of PRP on PDGFβ expression during wound healing.

### Histopathology findings

At 5 days, all groups revealed edema, necrosis and neutrophils infiltration with a covering cast. Re-epithelization was observed in ZnO NPs and PRP- ZnO NPs groups.

At 10 days, re-epithelization was observed at the periphery of the wound in PRP, ZnO NPs and lanolin groups. Furthermore, the inflammation subsided in PRP and PRP-ZnO NPs groups. Polymorphnuclear leukocytes infiltration was still observed in lanolin and PRP- lanolin groups. Granulation tissue was formed in all induced wounds but was well formed and organized in PRP group.

**Fig. 8:**
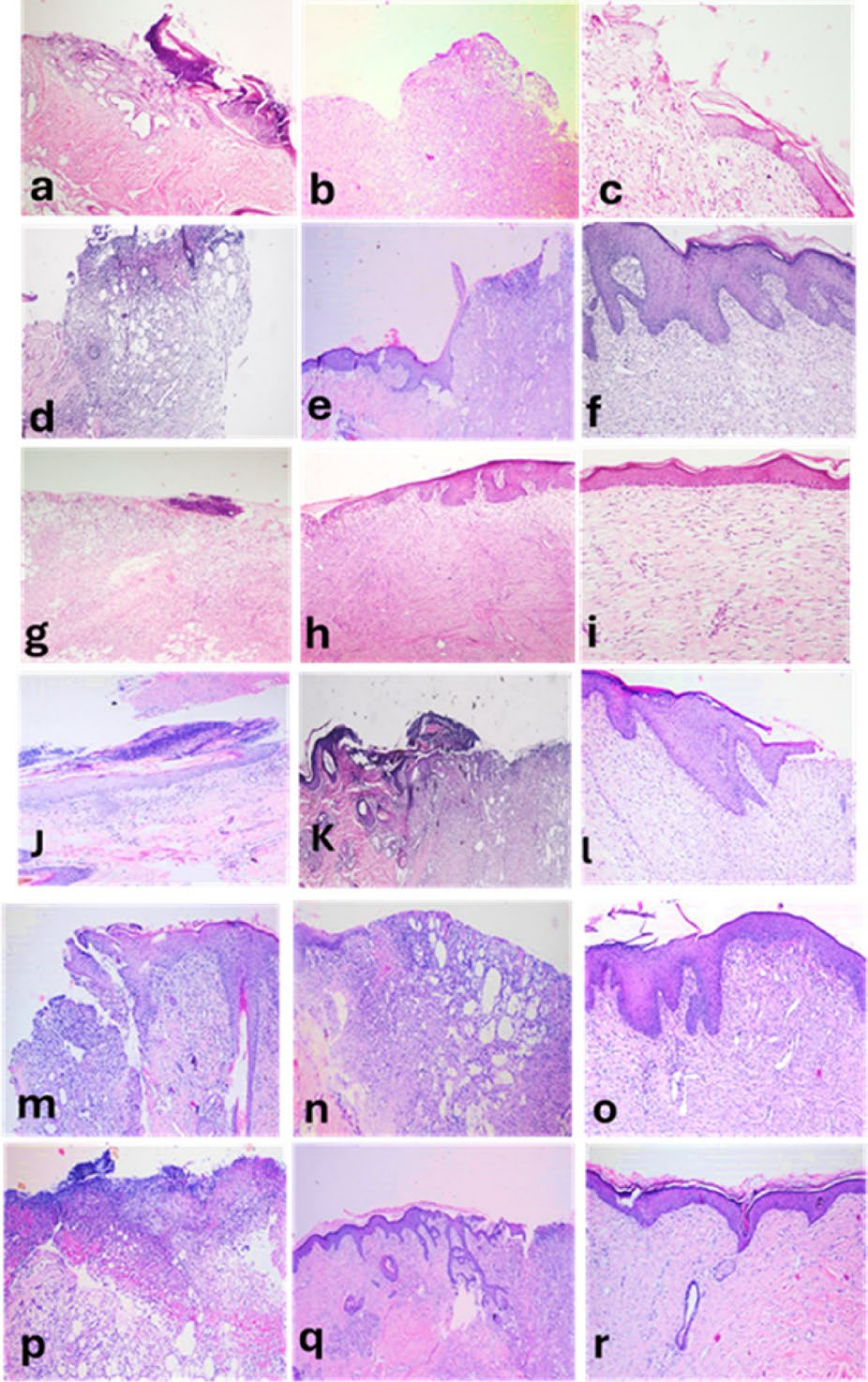
Histopathology of skin wound in different groups at 5-, 10- and 20-days post induction in dogs. Control wound at 5 days **(a)**, 10 days **(b)**, and 20 days **(c)**. Lanolin treated wound at 5 days **(d)**, 10 days **(e)** and 20 days **(f)**. PRP treated wound at 5 days **(g)**, 10 days **(h)**, and 20 days **(i)**. PRP-lanolin treated wounds at 5 days **(j)**, 10 days **(k)**, and 20 days **(l)**. ZnO NPs treated wound at 5 days **(m)**, 10 days **(n)**, and 20 days **(o)**. PRP-ZnO NPs treated wound at 5 days **(p)**, 10 days **(q)** and 20 days **(r)**. H and E stain. Magnification at 5 days and 10 days is 100 + X and 20 days is 200 X.

By 20 days, all groups showed epithelium bridging and granulation tissue formation. The epithelial thickness was consistent and well-formed. Granulation tissue was well-formed and well-organized in PRP and PRP-lanolin groups **(**Fig. 8**).**

**Fig. 9:**
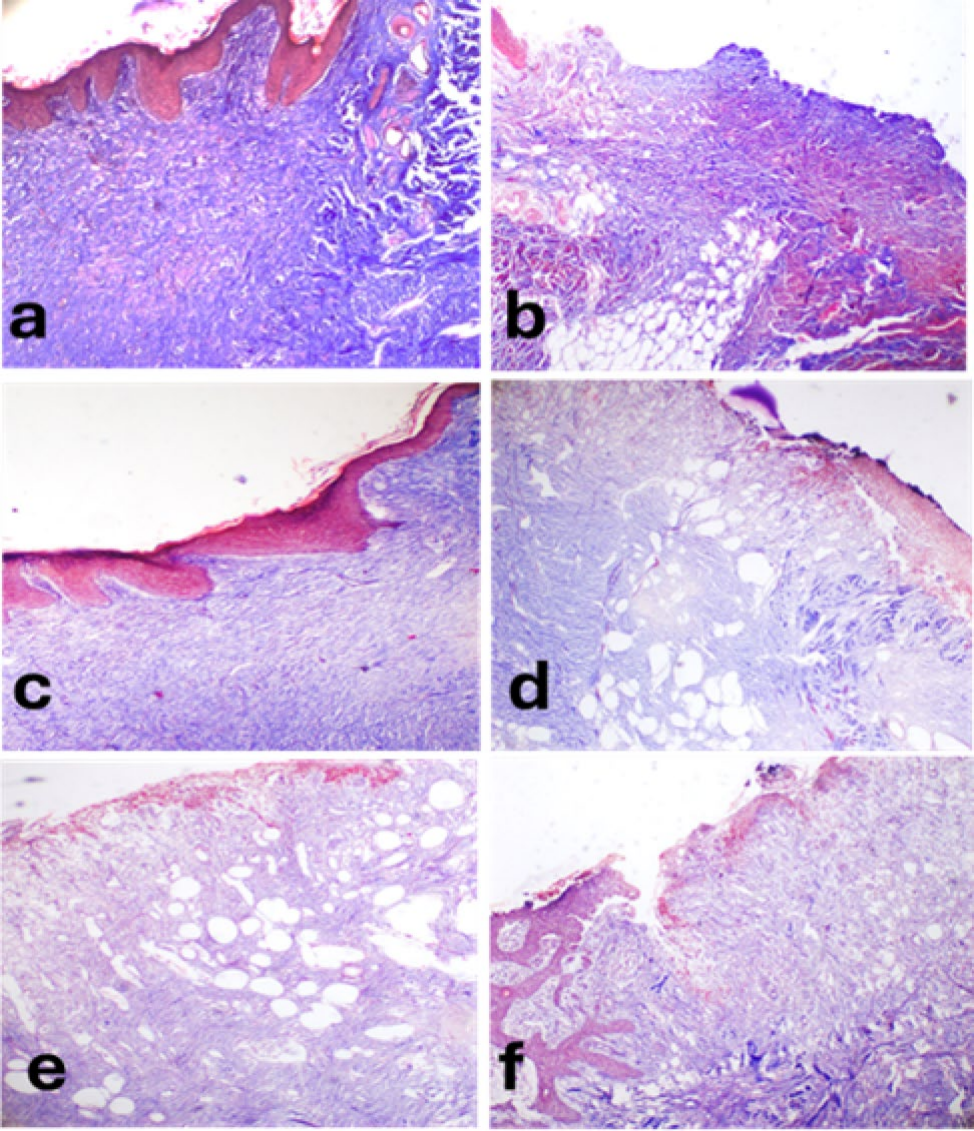
Skin wounds stained with Masson’s Trichrome in different groups at 10 days post induction. **(a)** The collagen bundles were disorganized with variation of color and infiltration in the control group. **(b)** The collagen bundles with parallel fibres in lanolin group. **(c)** The collagen bundles were organized and had parallel wavy fibres with consistent blue color in PRP group **(d)** The collagen bundles were organized, had parallel wavy fibres and breaks in parallel fibres in PRP- lanolin group. **(e)** Organized wavy parallel collagen bundles with breaks in ZnO NPs group. **(f)** The collagen bundles with little breaks in PRP-ZnO NPs group. MTC, X 100.

**Fig. 10:**
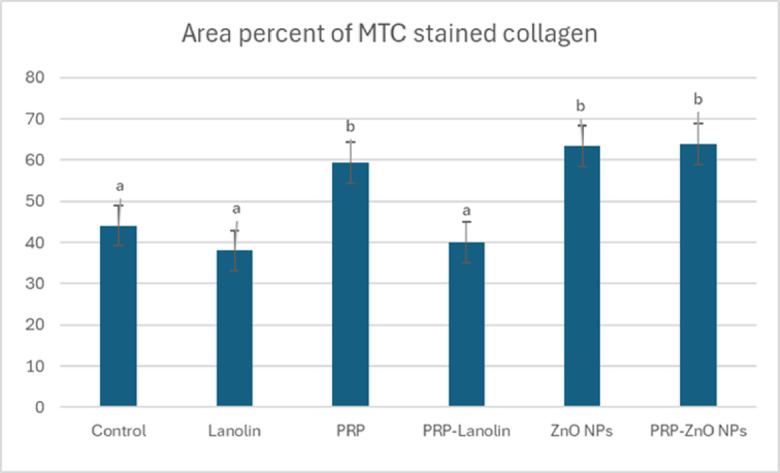
Mean area percent of MTC stained collagen in different groups at 10 days. The columns represent the mean area ± standard error. Columns bearing different lowercase letters are considered significant at *P*-value < 0.05.

MTC stained collagen was observed in the skin wounds. The collagen bundles were more organized with little variation in color in PRP, PRP-lanolin, ZnO NPs and PRP- ZnO NPs groups **(**Fig. 9**)**. The mean area percentage of MTC stained collagen was significantly elevated in PRP- ZnO NPs treated wound, ZnO NP and PRP groups compared to the control **(**Fig. 10**)**.

### Immunohistochemistry findings

**Fig. 11:**
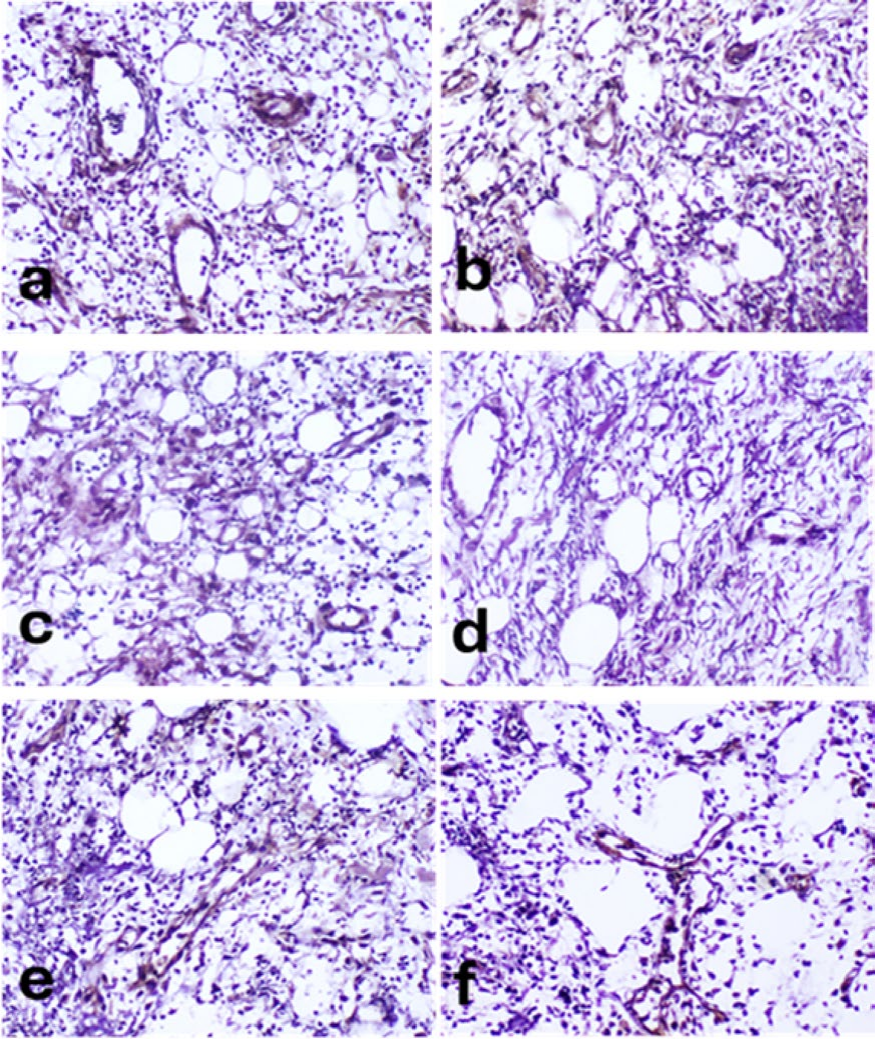
TNF- α immunohistochemistry of skin wounds in different groups at 5 days post induction showing: **(a)** Mild to moderate expression in the control group, **(b)** Moderate to severe expression in lanolin group, **(c)** Moderate expression in PRP group, **(d)** Mild to moderate expression in PRP- lanolin group, **(e)** Moderate expression in ZnO NPs group, **(f)** severe expression in PRP-ZnO NPs group. Immunoperoxidase, X400.

**Fig. 12:**
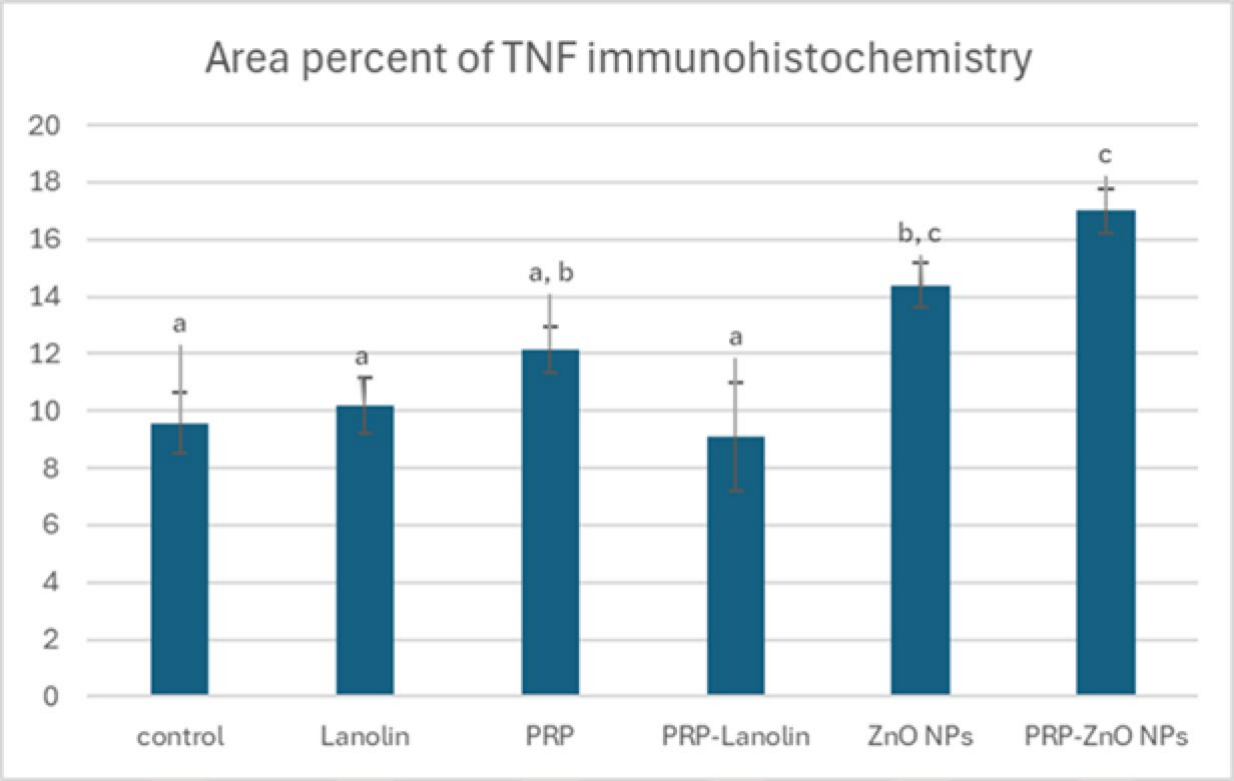
Mean area percentage of TNF- **α** immunohistochemistry in different groups at 5 days. The columns represent the mean area ± standard error. Columns bearing different lowercase letters were considered significant at *P*-value < 0.05.

TNF-α was expressed in the leukocytes infiltrating the wound and endothelial cells lining newly formed blood capillaries. It was mild to moderately expressed in control the group **(**Fig. 11a**)**, moderately to severely expressed in lanolin group **(**Fig. 11b**)**, moderately expressed in PRP group **(**Fig. 11c**)**, mild to moderately expressed in PRP-lanolin group **(**Fig. 11d**)**, moderately expressed in ZnO NPs group **(**Fig. 8e**)** and severely expressed in PRP- ZnO NPs group **(**Fig. 11f**)**. The mean area percent of TNF-α immunohistochemistry was significantly elevated in ZnO NPs and PRP- ZnO NPs groups compared to the control followed by PRP groups **(**Fig. 12**)**.

## Discussion

The treatment of wounds is being revolutionized by modern biology techniques such as gene therapy and bioengineered skin. This study evaluated the effects of topical infiltration of PRP and daily dressing with ZnO NPs ointment, both separately and in combination, on the healing of dogs’ cutaneous wounds. The integration of ZnO NPs and PRP in cutaneous wound healing represents a promising approach, leveraging their complementary properties. The present study demonstrated that PRP-ZnO NPs and PRP treatments markedly enhance wound healing timelines in comparison to the control wounds suggesting that ZnO NPs may prolong or stabilize PRP’s regenerative signaling. These findings align with the known antibacterial, antioxidant, and angiogenic properties of ZnO NP**s**^18^.

Animal models remain essential for evaluating wound healing therapies within a physiologically integrated environment that cannot be replicated in vitro. Dogs were chosen as an experimental model because of their physiological resemblance to human skin regeneration systems and their known importance in wound healing research. Similar healing processes, like as granulation tissue development and epithelialization, are seen in canine wounds and are crucial for translational research. Larger animal models offer more clinically relevant healing patterns than laboratory animal models like rodents, which heal mostly via contraction because of the panniculus carnosus^25^. Furthermore, the selection of mongrel dogs was intentional to minimize breed-specific genetic bias. Compared to purebred dogs, mongrel populations are more genetically diverse, which improves the findings’ external validity and generalizability^35^. Furthermore, the availability, affordability, and responsible usage of non-purpose-bred animals are all ethical and practical factors that support the use of mongrel dogs.

We intentionally used a within-animal experimental design in which each dog received all treatments. This approach is widely adopted in wound healing studies because it minimizes inter-individual variability (e.g., differences in immune response, genetics, metabolism, and skin characteristics). Also it allows each animal to act as its own control, thereby increasing statistical power and reduces the number of animals used, aligning with the principles of the 3Rs^36,37^.

Regarding systemic absorption and cross-treatment effects, we acknowledge the theoretical possibility of systemic absorption; however, the risk of meaningful systemic interference in our study is minimal due to the topical nature of the treatments, limited exposure area, and absence of clinically significant systemic effects during the study period. Moreover, the wounds were spatially separated, minimizing local diffusion or cross-contamination. In addition, previous studies using similar within-animal wound models have reported that systemic effects are negligible when treatments are applied topically and wounds are properly separated. In contrast, using one treatment per dog (between-animal design) would require a substantially larger number of animals, raising ethical concerns, introduce greater biological variability, and reduce statistical efficiency compared to paired or repeated-measures designs. Thus, the chosen design represents a balance between scientific rigor and ethical responsibility.

The PRP, rich in growth factors like PDGF and VEGF, promotes cellular proliferation, angiogenesis, collagen synthesis and essential to the wound healing cascade^38^. In a canine model, PRP enhances wound contraction and re-epithelialization^38,39^ while Iacopetti et al. showed that repeated PRP applications led to complete wound closure and improved healing^40^. Our study demonstrated similar outcomes with a single subcutaneous infiltration. Intralesional PRP also stimulates granulation tissue, collagen deposition, and vascularization as well as reduces inflammation^9,41^, supporting its role in superior healing with minimal scarring.

The ZnO NPs are well-recognized for their antibacterial, antioxidant, and angiogenic properties, all of which are critical in wound healing. Studies have demonstrated that ZnO NPs promote wound closure through enhanced re-epithelialization and collagen deposition^18^. For instance, Abbas et al. found that ZnO NPs ointment exhibited superior wound healing outcomes in animal models, with normal healing and no side effects^21^. Additionally, Li et al. highlighted the multifunctionality of ZnO/Ag biometallic nanofibers, which accelerated tissue regeneration and exhibited strong antibacterial effects^42^.

The role of ZnO NPs in stimulating angiogenesis and reducing oxidative stress has been particularly emphasized. Hydrogels incorporating ZnO NPs, not only enhanced biocompatibility but also accelerated wound healing in rabbits^43^. These findings are consistent across different animal models, suggesting that ZnO NPs have significant potential for therapeutic applications in veterinary wound care^44^. ZnO-NPs can exert bactericidal effects via the decomposition of the outer membranes of bacterial species by reactive oxygen species (ROS) and hydroxyl radicals (OH), which may lead to the peroxidation of phospholipid as well as the apoptosis of bacterial species. It has also been previously reported that ZnO NPs can be physically adhered to a bacterial wall to induce cell apoptosis^45–47^.

In our study, lanolin was utilized as a vehicle for nanoparticles, without playing a direct role in the wound healing process. This aligns with the findings of Chvapil et al., who demonstrated that lanolin primarily serves as a base or carrier for active agents, such as epidermal growth factor, and does not independently contribute significantly to wound healing^48^.

Weekly photographic documentation is a cornerstone of wound healing assessment, enabling consistent tracking of morphological changes such as contraction and granulation tissue formation. Standardized imaging protocols, including consistent angles and lighting, allowed for reproducible observations. Photographic methods have been widely utilized in studies, for instance, Sardari et al. employed serial images to evaluate wound healing progression in dogs treated with PRP, showing accelerated granulation and epithelialization^49^. Similarly, photographic analysis was used in studies on ZnO nanomaterial-based hydrogels, which demonstrated enhanced visual healing outcomes^19^.

Planimetry is a robust quantitative technique for measuring healing and re-epithelialization rates. By analyzing wound area reduction over time, planimetry provides a numerical representation of healing dynamics. Jee et al. demonstrated the effectiveness of planimetry in comparing PRP-treated wounds, revealing superior contraction and closure rates compared to controls^41^. In studies involving ZnO, planimetric tools were also critical for quantifying the rate of healing, with faster closure rates observed in ZnO nanomaterial-treated wounds^18^.

The efficacy of ZnO NPs, as demonstrated by the significant wound size reduction by day 21, is consistent with prior findings showing that ZnO NPs possess potent antimicrobial, antioxidant, and anti-inflammatory properties, which collectively create a conducive environment for wound healing^50,51^. Their nanoscale size allows for better cellular interaction and penetration into tissues, enhancing antibacterial efficacy and reducing infection risk key impediments in chronic wound healing^5^.

Most notably, the combination therapy of PRP–ZnO NPs produced the most profound effects, significantly outperforming individual treatments and control groups at all-time points. This suggests a synergistic effect, where PRP promotes regenerative signaling while ZnO NPs reduce oxidative stress and microbial load. Similar synergistic outcomes have been documented in composite biomaterials combining bioactive molecules and nanoparticles^52^.

All treatment groups showed progressive wound contraction over time, with PRP–ZnO NPs, PRP and ZnO NPs groups outperforming control and lanolin at every time point (*P* < 0.001). This significant wound contraction enhancement reflects the combined effects of tissue regeneration by PRP and antimicrobial action by ZnO NPs. These findings are consistent with prior work demonstrating that PRP accelerates fibroblast proliferation, granulation tissue formation, and extracellular matrix remodeling^39^. Similarly, ZnO NPs have been shown to support rapid wound closure through their antibacterial, antioxidant, and anti-inflammatory properties^50,51^.

The healing percentage followed a similar trend, with statistically significant differences between groups (*P* < 0.001) and across time points. By day 21, nearly complete healing was observed in PRP–ZnO NPs (99.46 ± 0.32%), ZnO NPs (99.44 ± 0.41%), and PRP (99.31 ± 0.38%) groups. These findings confirm the superior regenerative efficacy of active treatments over control. Prior studies have noted similar near-total wound healing using ZnO nanofibers and PRP-based therapies^18,52^.

Epithelialization that is crucial for re-establishing the skin barrier was significantly higher in all treatment groups than in control (*P* < 0.001). By day 21, PRP, ZnO NPs and PRP–ZnO NPs showed the best epithelial coverage. These results highlight the accelerated re-epithelialization promoted by bioactive and nanomaterial-based interventions. This is in agreement with previous studies suggesting that, ZnO NPs enhance epithelial regeneration by reducing microbial burden and stimulating keratinocyte migration, while PRP supports epithelial cell proliferation through PDGF and VEGF content. PRP’s direct action on basal epithelial cells has been documented in studies involving both skin and mucosal wound model^51^. Although PRP-lanolin showed moderate improvement in epithelialization and contraction, it was consistently less effective than PRP-ZnO NPs, reinforcing the superior role of ZnO NPs over passive emollients.

Oxidative stress plays a key role in delaying wound healing, making antioxidant activity a crucial aspect of therapeutic efficacy. This study measured TAC and MDA to evaluate redox dynamics during wound healing across various treatment groups^53^. Significant time-dependent changes in both parameters were observed, particularly in the PRP-, ZnO NP-, and PRP–ZnO NP-treated groups.

At baseline (day 0), TAC levels were similar across all groups, indicating no inherent differences in systemic oxidative stress. However, by day 5, significant reductions in TAC were seen in all treatment groups compared to control, with PRP and PRP–ZnO NPs showing the lowest values. These lower TAC values suggest higher antioxidant consumption at the wound site, reflecting active scavenging of ROS generated during early tissue remodeling^50,51^.

By day 10 and continuing to day 20, TAC levels remained significantly lower in the PRP, ZnO NPs and PRP–ZnO NPs groups compared to lanolin and control groups. This sustained depletion suggests ongoing antioxidant use during intensive cell proliferation and matrix synthesis. These patterns are consistent with previous findings showing that bioactive agents accelerate healing by enhancing redox turnover at the wound site^5^. The combinations of PRP with ZnO NPs or lanolin showed intermediate TAC values, suggesting a potential balancing effect between pro-regenerative and antioxidative roles^54^.

MDA is a biomarker of lipid peroxidation and oxidative stress. Assessment of tissue MDA revealed that elevated MDA levels indicate increased oxidative stress, which can impair tissue regeneration and delay healing^53^. The results demonstrated that skin wounds incorporating PRP, particularly when combined with ZnO NPs or lanolin, effectively mitigate oxidative stress during wound healing. Reducing MDA levels is critical, as oxidative stress can delay healing by damaging cell membranes, proteins, and DNA^55^.While all groups had comparable levels at baseline, by day 5, the PRP–ZnO NPs group exhibited a remarkable reduction in MDA, outperforming PRP, ZnO NPs and lanolin, with the control group maintaining the highest levels. This trend continued over time, with day 10 and day 20 data showing the most dramatic decreases in MDA in PRP–ZnO NPs and PRP, indicating reduced oxidative damage and improved healing microenvironments. This is in agreement with earlier studies^56,57^.

The MEPE is one of the “matricellular protein” related to SIBLING family (small integrin-binding ligand, N-linked glycoproteins family**)**^58,59^. Though they can attach to structural ECM elements like collagen fibrils or basement membrane, matricellular proteins are released into the ECM and are thought to have no role in their mechanical activities^60^. In addition, it is strictly controlled to fine-tune its roles during tissue healing and maintenance^61^. In the adult homeostatic tissues, their expression levels drastically decline. Nonetheless, as tissue damage, inflammation, cancer, and other diseases heal, the expression of certain matricellular proteins is triggered^62^.

MEPE is associated with ECM remodeling and mineralization. At all post-treatment intervals, the PRP–ZnO NPs group demonstrated the highest MEPE expression, particularly by day 5, indicating strong stimulation of matrix regeneration. PRP alone also induced significant MEPE expression, with consistently higher levels than the control and ZnO NPs alone. The expression of MEPE collectively demonstrated superior regenerative potential of PRP, particularly when combined with ZnO NPs. The sustained upregulation of MEPE in the PRP and PRP-ZnO NPs groups indicates enhanced ECM synthesis and possibly accelerated wound healing. The synergistic effect observed with PRP-ZnO NPs suggests that ZnO NPs may augment the biological activity of PRP, potentially through its antimicrobial and anti-inflammatory properties, creating a favorable environment for tissue repair^18^.

The TGF-β is a pivotal cytokine in the wound healing cascade, known for regulating inflammation, stimulating fibroblast activity, and promoting ECM deposition^63^. The obtained results confirm that PRP and PRP-ZnO NPs treatments markedly enhance TGF-β expression throughout the wound healing timeline, particularly in the proliferative and remodelling phases. This in agreement with previous studies whom suggesting the role of PRP in enhancing fibroblast proliferation, collagen synthesis, and granulation tissue formation and increasing the expression of TGF-β in rat burn injury^64–66^. Conversely, ZnO NPs alone have limited impact on TGF-β expression unless paired with other bioactive factors. The moderate expression seen in the PRP–lanolin group suggests that emollient carriers may aid growth factor retention but do not intrinsically amplify cytokine signaling or regenerative pathways.

The most significant upregulation of TGF-β was observed in the PRP group across all time points, with a peak at day 10. PRP–ZnO NPs showed moderate upregulation but less than PRP alone, suggesting that while combination therapy benefits structural healing (as seen with MEPE), PRP alone is more potent in activating TGF-β pathways.

The TNF-α is a pro-inflammatory cytokine plays a crucial role in the early process of wound healing in skin, its suppression is critical to transition from inflammation to proliferation^67^. All treated groups showed a significant reduction in TNF-α levels over time (*P* ≤ 0.001). PRP achieved the most consistent suppression, especially by day 20, reflecting strong anti-inflammatory activity. ZnO NPs also significantly reduced TNF-α by promoting antioxidant and antimicrobial defense^68^.

Surprisingly, the PRP–ZnO NPs combination, while effective in promoting MEPE and wound contraction, was less effective than PRP or ZnO NPs alone in reducing TNF-α levels at later stages. This suggests that while the combination supports regeneration, it may dampen some anti-inflammatory effects of the individual components. A similar antagonistic interaction was observed in a study by Ramachandran et al., where mixed nanocomposite therapies showed non-additive cytokine suppression^69^. The consistently higher TNF-α levels in control and lanolin groups across all time points highlight the limited ability of these treatments to resolve inflammation, explaining their poorer wound healing outcomes.

The PDGFβ is a vital growth factor involved in multiple phases of wound healing, particularly by stimulating fibroblast proliferation, angiogenesis, and ECM remodeling essential for tissue repair^38^. In this study, PDGF-β expression increased significantly over time across all groups, but the magnitude and consistency of this increase varied depending on the treatment. The most substantial and sustained elevation of PDGFβ was observed in the PRP and PRP–ZnO NP groups, confirming the regenerative potency of platelet-derived therapies. PRP is inherently rich in PDGFβ and releases it upon activation, creating a bioactive environment that supports robust tissue regeneration. These elevated PDGFβ levels reflect PRP’s natural concentration of growth factors, which play a central role in promoting cell proliferation, angiogenesis, and ECM deposition^70^. Experimental studies further demonstrate that PRP significantly increases PDGFΒ levels in skin tissue and enhances histological indicators of healing in wound models^71^. This supports the hypothesis that PRP creates an enriched microenvironment favorable for tissue repair and regeneration^72^.

The PRP–ZnO NP combination also produced consistently high PDGFβ levels, slightly lower than PRP alone but still markedly higher than other groups. This suggests a supportive role of ZnO NPs in maintaining a wound microenvironment conducive to sustained PDGFβ activity likely due to their antimicrobial, antioxidant, and anti-inflammatory properties. While ZnO NPs alone did not significantly elevate PDGFβ expression, their presence may protect and prolong the functional activity of growth factors within the wound milieu^51^. The PRP–lanolin group also demonstrated increased PDGFβ expression, though to a lesser extent. This may reflect some enhancement in growth factor retention due to the occlusive nature of lanolin, but without the biochemical synergy seen in the PRP–ZnO NP group. In contrast, control, lanolin, and ZnO NP-only treatments resulted in minimal PDGFβ expression over time. This indicates that without a direct biological stimulant like PRP, these treatments lack the capacity to initiate or sustain the signaling pathways necessary for optimal fibroblast activation and matrix production.

Histologically, wounds treated with PRP and ZnO NPs exhibited more rapid and organized re-epithelialization, substantial granulation tissue formation, and better collagen fiber organization compared to controls. These effects align with the known regenerative and angiogenic properties of PRP, which releases growth factors such as PDGF, VEGF, and TGF-β to stimulate fibroblast proliferation and matrix deposition^38^. The addition of ZnO NPs appears to enhance these outcomes by promoting antimicrobial activity, cellular proliferation, and oxidative stress modulation^73^.

At the immunohistochemical level, TNF-α expression was significantly elevated in wounds treated with ZnO NPs and PRP. While TNF-α is a pro-inflammatory cytokine typically associated with early wound healing, sustained yet controlled expression can be beneficial by facilitating immune cell recruitment and angiogenesis. This controlled elevation observed here suggests that ZnO NPs contribute to a balanced inflammatory response, supporting timely progression through the inflammatory phase of healing^74^.

Collagen deposition, a crucial indicator of tissue remodeling, was markedly increased in PRP and ZnO NP-treated groups. Enhanced MTC staining indicated higher collagen content and more organized fiber alignment, particularly in the ZnO–PRP combination group. This observation is consistent with previous studies showing that ZnO NPs stimulate fibroblast activity and collagen synthesis^5^, while PRP further contributes through TGF-β1 signaling pathways^73^.

A potential limitation of the present study may be the application of multiple treatment modalities within the same animal, which may introduce a degree of intra-subject correlation and limit complete biological independence among experimental wounds. Nevertheless, the study was intentionally designed as a within-subject comparative model to minimize inter-individual variability and to ensure maximal standardization of physiological, environmental, and surgical conditions across treatment groups. Furthermore, cutaneous wound healing is primarily governed by local paracrine and autocrine mechanisms, while the systemic dissemination of topically applied agents, particularly nanomaterials and PRP-derived products, is considered minimal. Although minor systemic influences cannot be entirely excluded, any such effects would likely occur uniformly across all wounds within the same subject and therefore would be unlikely to substantially confound the comparative interpretation of treatment outcomes. Accordingly, the findings should be interpreted within the context of a controlled preclinical experimental model. Future studies employing fully randomized independent-subject designs with larger sample sizes are warranted to further validate and extend the present findings.

## Conclusions

The findings of this study demonstrated that both PRP infiltration and ZnO NPs ointment independently enhance wound healing through distinct mechanisms. PRP infiltration demonstrated the most effective enhancement of wound healing, as evidenced by the highest levels of key regenerative growth factors (TGF-β and PDGFΒ) that promoted tissue regeneration by delivering concentrated growth factors directly to the wound site, thereby accelerating epithelialization and collagen deposition. Similarly, ZnO NPs ointment, when applied as a daily dressing, provided potent antimicrobial properties and supported cellular proliferation and angiogenesis, contributing to improved healing outcomes. However, the combination of PRP with ZnO NPs (PRP–ZnO NPs) exhibited strong therapeutic benefits, particularly in modulating oxidative stress and inflammation, as reflected by reduced MDA and TNF-α levels. Although PRP–ZnO NPs did not surpass PRP in growth factor expression, it provided consistent healing acceleration across parameters. These findings highlight both PRP and PRP–ZnO NPs as effective wound healing interventions, with PRP–ZnO NPs offering added anti-inflammatory and oxidative benefits that support their synergistic use in advanced skin repair strategies.

## Data Availability

All data used and/or analyzed during this research are available from the corresponding author on reasonable request.
